# A personalized communication efficient federated learning framework with low rank adaptation for intelligent leukemia diagnosis

**DOI:** 10.1038/s41598-025-29672-1

**Published:** 2025-12-11

**Authors:** P. Suresh, P. Keerthika, A. R. Nitesh Kumar

**Affiliations:** School of Computer Science and Engineering, Vellore Institute of Technology, Vellore, India

**Keywords:** Medical imaging, Federated optimization, Blood cancer, Communication-efficient learning, Explainable AI, Cancer, Computational biology and bioinformatics, Health care, Mathematics and computing

## Abstract

Leukemia diagnosis with medical imaging necessitates the development of highly accurate and individualized models that uphold data privacy among institutions. This research proposes a framework named FedPerLoRA-Health, a communication-efficient federated learning framework that combines federated personalization and low rank adaptation with EfficientNet architectures for personalized leukemia detection. The proposed PerFLR-EffNet algorithm holds the structural efficiency of EfficientNet variants B0 and B2 as backbone models, facilitating parameter-efficient updates and local personalization across diverse client datasets. Within this framework, personalized layers undergo local training, whereas LoRA-adapted global layers are disseminated to reduce communication overhead. The proposed method is assessed on a Blood Cells Cancer Acute Lymphoblastic Leukemia (ALL) dataset with classification-based metrics such as accuracy, precision, recall and F1-score and federated learning-based metrics such as communication cost and convergence rate. The efficiency of the proposed model is analysed by comparing it with the baseline models such as centralized EfficientNetB0 and EfficientNetB2 without personalized Federation. Experimental results indicate that PerFLR-EffNet attains a better average classification accuracy of 98.67% and also proves to be communication efficient by reporting reduced number of trainable parameters and a reduction in communication overhead by 88.12% when compared with the baseline models.

## Introduction

One of the most prevalent and severe blood cancers in children and adolescents is Acute Lymphoblastic Leukemia (ALL). Better treatment and survival rates depend on early and precise diagnosis. Traditional diagnosis includes experienced pathologists manually examining blood smear pictures, which is time-consuming, resource-intensive, and subject to observer variability. Haematological cancer detection has adopted artificial intelligence (AI), notably deep learning (DL)-based image classification approaches, to meet the need for fast, accurate, and scalable diagnostics^1^. Modern lightweight convolutional neural networks (CNNs) like EfficientNet have shown promising results in medical picture categorization due to their high accuracy-to-parameter ratio. EfficientNet variants B0 and B2 can process pictures locally without internet connectivity or significant server infrastructure, making them ideal for edge devices^2^. However, traditional centralized training of such models presents data privacy problems, especially in medical settings where GDPR and HIPAA protect patient data.

Federated Learning (FL) is a promising option that allows AI model training across decentralized data silos without transmitting raw data to a central server. For data sovereignty and privacy compliance, each institution (or client) trains a local model and only updates the central server^3^. Despite these benefits, FL techniques like FedAvg suffer various real-world deployment obstacles such as data heterogeneity and communication bottlenecks**.** Clinical data is non-IID due to demographics, acquisition processes, and staining techniques varying among medical institutions. Global models may underperform local client data due to heterogeneity^4^. Also, frequent model synchronization in FL can cause bandwidth constraints, particularly for big models relayed across restricted edge networks^5^. Standard federated techniques lack in personalization that makes it less efficient at specific clients^6^.

In order to bridge these gaps which are identified with the standard federated approaches, this research work aims to develop and evaluate FedPerLoRA-Health, a unique, communication-efficient and personalized federated learning system for accurate leukemia diagnosis from medical images in decentralized clinical settings. FedPer (Federated Personalization) supports client-specific adaptation^7^ and Low-Rank Adaptation (LoRA) minimizes communication overhead^8^ during model updates and EfficientNet variants B0 and B2 provide efficient and high-quality feature extraction. EfficientNet B0 and B2 variants were chosen as their higher variants might directly counteract the primary objective of the proposed work and results in high communication overhead and demands high computation which makes it less suitable. Using these components, this research addresses real-world federated medical learning difficulties such as avoiding centralized data aggregation and handling heterogeneous medical institution data distributions. This helps in protecting the privacy of patients and reducing edge client communication. The novelty of this work relies in providing dual personalization mechanism where only the personalized adapter updates is shared with the global server and not model weights. This makes it different from simple integration of FerPer, which sends full model updates, LoRA which trains a single, global model and EfficientNet base.

Key contributions of this research include the following:

- A personalized framework, FedPerLoRA-Health facilitating client-specific personalization with layered separation provided by FedPer and LoRA based parameter efficient updates to maintain accuracy while drastically reducing parameter transmission.
- A novel federated learning algorithm, PerFLR-EffNet, integrates federated personalization with Low-Rank Adaptation and EfficientNet (B0 and B2) to achieve high diagnostic performance while preserving privacy across diverse clients.
- A communication efficient federated approach to reduce communication overhead by transmitting only minimal model updates with limited network bandwidth for bandwidth-constrained clinical environments.

## Related work

Recent advances in artificial intelligence, edge computing and federated learning have improved medical diagnosis, particularly for hematopoietic malignancies such as Acute Lymphoblastic Leukemia. Many studies have examined deep learning-based ALL classification and privacy-preserving machine learning algorithms for collaborative model training that does not compromise patient data. Meanwhile, personalized federated learning algorithms and parameter-efficient fine-tuning approaches like LoRA have gained popularity as answers to dispersed environments’ non-IID data distributions and communication restrictions. This section reviews research in four key areas: ALL detection using deep learning architectures, federated learning applications in medical imaging, personalized federated frameworks such as FedPer and pFedMe, and LoRA for lightweight model adaptation. This underscores the research gap of not having a cohesive solution that integrates EfficientNet, FedPer, and LoRA in a federated configuration for ALL prediction. A Novel Deep Learning Segmentation and Classification Framework used custom UNet for leukemic cell segmentation and downstream classification^9^.

Deep learning has transformed automated hematologic oncology diagnosis, especially with blood smear images for ALL dataset. CNN-based models are used to extract high-level features from microscopic images ^10^. EfficientNet versions with compound scaling and higher parameter efficiency perform well in medical imaging. Das and Meher created an EfficientNet-based classifier for ALL diagnosis that outperformed CNNs like AlexNet and ResNet with over 99% accuracy^11^. Rahman et al. found that ResNet50 along with nature inspired algorithms for feature selection outperformed other CNN architectures in terms of blood cancer classification accuracy and processing efficiency^12^. Recent research confirms that EfficientNet-B3 handles class imbalances improving the model complexity and accuracy, making it suitable for mobile and edge healthcare devices^13^. A lightweight EfficientNet-B3 model is proposed for classifying ALL with less trainable parameters^14^. According to a recent study ^15^, EfficientNet variants perform better in medical image classification tasks owing to their compound scaling method and lower processing complexity. Elsayed et al. examined bone marrow image-based deep learning models for ALL diagnosis through 2023. In several circumstances, specialized CNNs with 100% accuracy showed strong classification performance when boosted using boosting methods and optimization strategies^16^.

FL is a decentralized machine learning method where clients train a global model without sharing data. This benefits privacy-sensitive industries, such as healthcare. In their comprehensive assessment of FL in medical imaging, Sohan et al. stressed its capacity to protect privacy while facilitating clinical institution-wide model construction^3^. Another study found that PPPML-HMI, a tailored FL framework for heterogeneous medical imaging, increases model robustness and privacy protection across institutions^17^. Traditional FL frameworks perform poorly with non-independent and identically distributed (non-IID) client data and high communication costs, making them unsuitable for healthcare^18^. A comprehensive MDPI survey^19^ highlighted FL’s applicability of FL in CT, MRI, and histopathology. F. Zang et.al demonstrated that FL works in resource-constrained medical situations with minimal data and annotation^20^.

Mazid et al. introduced blockchain-driven customized federated learning for the Internet of Medical Things to provide decentralized trust, data security, and efficient model training across distributed medical devices^21^. Sharma et al. proposed a blockchain-enabled federated learning system for privacy-preserved electronic health records that protects sensitive patient data and supports collaborative model building across many institutions^22^. Both studies show how blockchain and federated learning can help make healthcare AI systems secure, transparent, and privacy-preserving.

Several Personalized Federated Learning (PFL) frameworks have been developed to address the shortcomings of FL in non-IID conditions. A novel method, pFedLT performs layer-wise transformation to capture the diversity of data distribution among clients^23^. A bi-level optimization target based on Moreau envelope regularization balances global knowledge and local adaptation in pFedMe^24^. PFL is expanded by the PFed-NS framework^25^, which dynamically reconfigures neural structures to meet the client’s needs. This architecture is important in healthcare, where inter-institutional variance is widespread. An improved version of FedPer shows improvement in personalization both in terms of accuracy and stability^26^. For smoother personalization, pFedMe uses Moreau envelope regularization and converges with significant data heterogeneity. These methods move away from “one-size-fits-all” FL models toward client-adaptive learning architectures.

Some customized FL approaches handle client heterogeneity without full model update. Local batch-normalization layers in FedBN prevent feature distribution shifts among clients, increasing convergence in non-IID circumstances^27^. FedAMP employs an attention-based message-forwarding mechanism, allowing clients to prioritize updates from comparable peers and personalize them based on inter-client affinities^28^. FedRep allows personalization with minimum communication of shared features by learning a shared backbone globally, while each client maintains its own classifier head ^29^. FedPerLoRA-Health freezes the majority of the EfficientNet backbone and personalizes it via lightweight LoRA adapters, lowering communication and computation while enabling fine-grained medical diagnosis client adaptation.

LoRA is a lightweight fine-tuning strategy that inserts trainable rank-decomposed matrices into frozen model weights, thereby lowering the number of adjustable parameters. LoRA, popularized for large language models, is becoming increasingly popular in vision-based applications. LoRA was applied to large vision models for lung nodule detection, attaining near-state-of-the-art accuracy with only 3% parameter updates^30^. In another study, RadLoRA improved latency and accuracy for real-time radiological image classification, which is critical for healthcare edge AI^31^. These studies demonstrate the potential of LoRA for communication-efficient federated training. Computerized Medical Imaging and Graphics reported a LoRA-enhanced RT-DETR for musculoskeletal ultrasound segmentation with quick convergence and fewer trainable parameters than the previous model^32^. This strategy works for federated learning, where communication and memory efficiency are important. A summary of these related works are provided in Table 1.

**Table 1:** Summary of Existing Methods.

S.No	Methodological Insights	Strengths	Limitations
1	Leukemia segmentation + classification with DL ^9^	High diagnostic precision	Centralized DL The lack of customization and FL support Limits hospital scalability
2	CNN for ALL detection ^11^	High classification accuracy	Model trained on single dataset fails on non-IID cross-site data
3	Multiclass CNN with optimal characteristics ^12^	Treats multiple blood cancers	Feature optimization cannot handle dispersed data shifts
4	Transfer learning for leukemia detection ^13^	Improves limited data performance	Transfer learning alone cannot personalize federated systems
5	Lightweight EfficientNetB3 ^14^	Low latency, efficient	No FL integration Unsuitable for non-IID medical data distribution
6	DL on bone marrow images ^16^	Clinically relevant method	No communication- efficient FL hospital collaboration methods in practice
7	PPPML-HMI for FL heterogeneity ^17^	Strong customization + privacy	High computation cost No lightweight LoRA adaption
8	FL in radiology ^18^	Includes real-world examples	Limited customization, Generic FL techniques
9	Meta-learning for personalized FL ^23^	Improves local adaptability	Meta-learning is resource-intensive and unsuitable for low-resource healthcare nodes
10	FL Moreau envelopes ^24^	A strong theoretical contribution	Computationally expensive Unsuitable for large-scale medical imaging
11	Neural segmentation for PFed-NS ^25^	Adaptive personalization	Complex segmentation makes hospital deployment impractical
12	FedPer + + for non-IID data ^26^	Enhances personalization	Communication-heavy No LoRA-like lightweight adaption
13	Classifying lung nodules with LoRA ^30^	Effective LoRA adaption	Centralized imaging No federated personalization
14	RadLoRA for radiology ^31^	Low-rank tweaking increases efficiency	Task-specific Not applicable to personalized FL
15	LoRA-enhanced ultrasound DETR ^32^	Real-time detection	Not intended for federated or personalized healthcare learning
16	FedBN with local BN ^27^	Increases resilience to non-IID	Lacks efficient lightweight low-rank adaption
17	Shared representations in FL ^29^	Balances global and local	Communication heavy Non-optimized for LoRA/EfficientNet

EfficientNet has been extensively researched for ALL detection, and Federated Learning and Personalized Federated Learning frameworks, such as FedPer and pFedMe, have shown success with heterogeneous client data. However, LoRA for parameter-efficient adaptation within a personalized federated framework for leukemia prediction has not been studied. No previous work has combined EfficientNet, FedPer and LoRA in a leukemia diagnostics-specific federated architecture. Hence, this paper integrates FedPer, LoRA and EfficientNet variants to detect leukemia. The proposed approach builds on EfficientNet, FL and LoRA, but differs from personalized or communication-efficient FL techniques in several aspects. FedProx reduces statistical heterogeneity with a proximal term and pFedMe personalizes clients with Moreau envelope regularization. Both strategies require the updating and transmission of large model parameters, which increases the communication overhead. FedPerLoRA trains and communicates lightweight LoRA adapters while freezing the EfficientNet backbone, thereby decreasing computational and communication expenses. The proposed method uses EfficientNet to exploit strong feature extraction for medical imaging and systematically evaluates the trade-off between personalization and communication efficiency in leukemia diagnosis. Other LoRA-FL approaches mostly demonstrate parameter-efficient adaptation in general FL settings. Thus, FedPerLoRA-Health offers a domain-specific, communication-efficient, and tailored FL framework that surpasses FedProx, pFedMe, and other LoRA-FL techniques.

## Materials and methods

This section begins with the entire research workflow, from data partitioning to model evaluation. Next, EfficientNet-B0 and B2 backbone networks were described for efficient and scalable medical picture analysis. The integration of LoRA modules in EfficientNet layers is detailed to enable parameter-efficient learning. In the next subsection, the personalized local training for each federated client is detailed. Finally, the client–server communication loop and FedAdam optimizer for client update aggregation are presented in the federated-training process. This method was formalized in the final algorithm.

### Architecture and process flow of the FedPerLoRA-Health framework

**Fig. 1:**
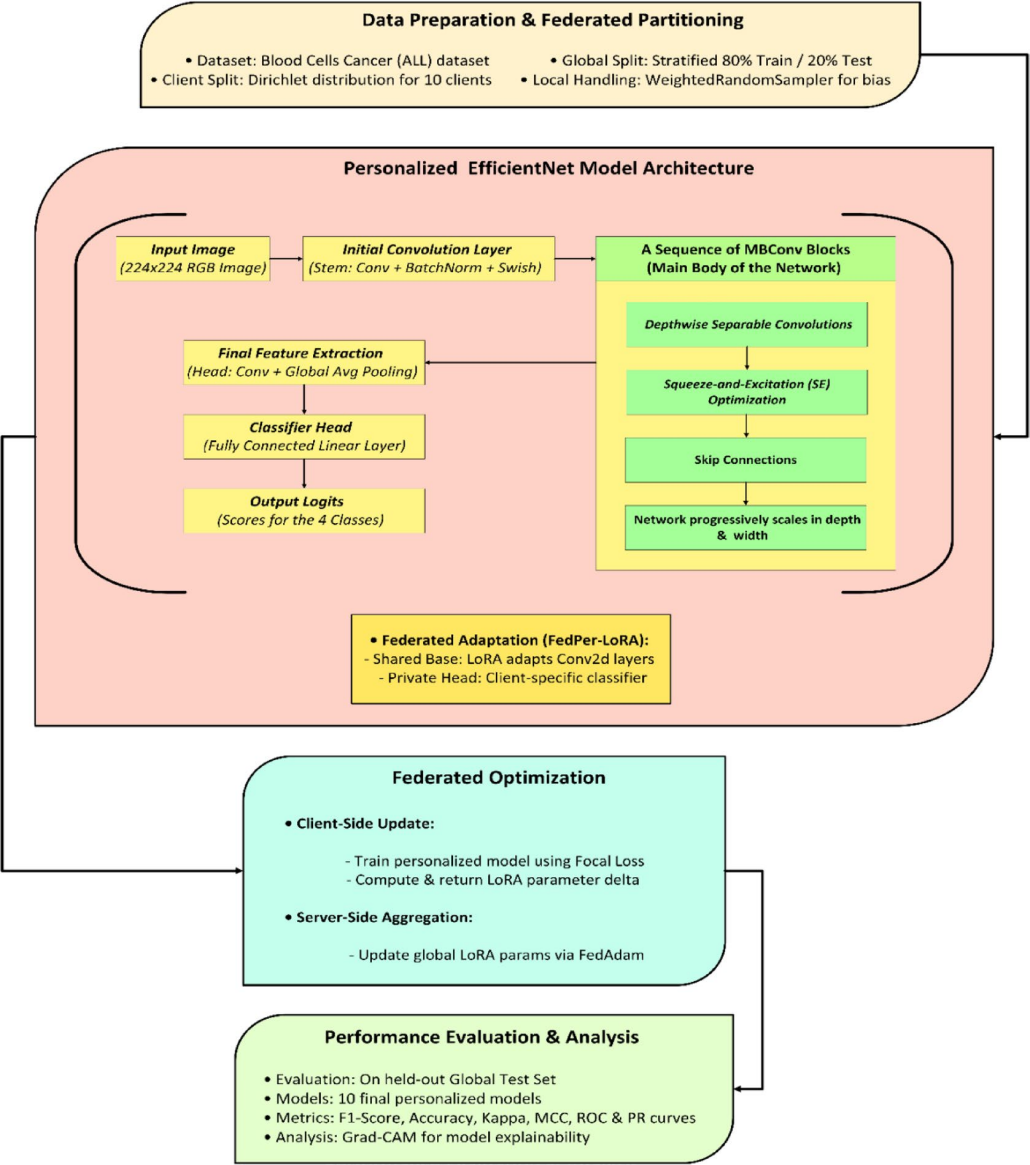
Architecture and Process Flow of the FedPerLoRA-Health Framework.

The architecture and process of FedPerLoRA-Health for efficient and personalized federated learning in blood cancer classification from microscopic blood smear images are shown in Fig. 1. The process begins with data partitioning and preparation. The Dirichlet distribution simulates non-IID data across 10 clients using 80% training and 20% testing samples from the global dataset. All RGB images are pre-processed to 224 × 224 resolution. The initial convolutional layer, batch normalization, and swish activation uses this tensor, and the timm library scales feature maps for EfficientNet-B2 as 260 × 260 input. All clients has its own lightweight LoRA module in the convolutional layers. Each client receives data distribution-specific classification head. This allows for efficient and private personalization. All clients trained their models using class imbalance resistant focus loss functions. After local training, only the LoRA module delta parameters were communicated to the server. These local deltas are collected by the FedAdam optimizer on the server side, updating the global LoRA parameters while maintaining the common base model. FedAdam is preferred because of its enhanced convergence speed and stability, which makes it desirable in heterogeneous federated setups. Unlike FedAvg, which utilizes simple averaging and is unstable with non-IID data, FedAdam reduces the gradient variance between clients by using adaptive learning rates and momentum-based updates. FedAdam optimizes faster and smoother by dynamically altering global updates based on historical gradients than FedProx, which adds a proximal term to control client drift but slows the convergence.

In this process flow, the ConV2D layers were shared with the global server and the Classifier Head is personalized at each client. Multiple federated rounds of client–server communication are continued. The final performance of the framework was tested on a held-out global test set. Each of the 10 clients received a customized model, which was evaluated using the F1-score, accuracy, Cohen’s Kappa, MCC, ROC, and PR curves. Grad-CAM visualizes class-specific attention in input photos to improve the model explainability.

### FedPerLoRA-Health framework

The proposed FedPerLoRA-Health framework integrates FedPer for personalizing, LoRA adapters for communication efficiency, and EfficientNet backbones for lightweight deep learning to train models on non-IID blood smear images from hospitals without sharing raw data. Each client retains its private, non-IID blood smear dataset and trains only lightweight LoRA adapters and a private classification head on a frozen EfficientNet backbone (B0/B2) to address class imbalance using focal loss and weighted sampling.

**Fig. 2:**
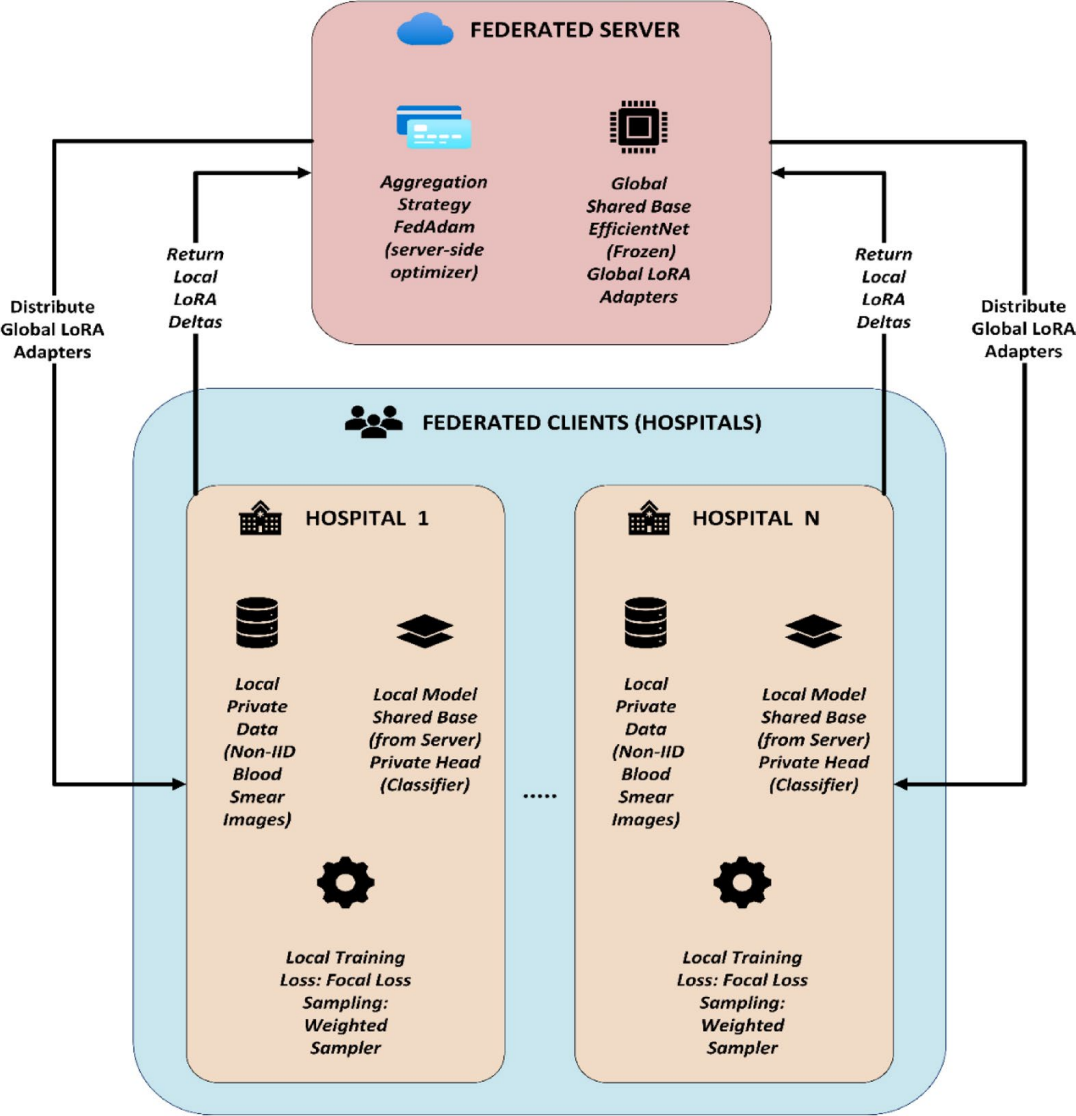
Customized Model Training Process with Shared Base.

The federated server manages the shared EfficientNet base and global LoRA adapter. Each communication round exchanges solely LoRA adapter deltas; clients receive global adapters, train locally, and return updates that FedAdam collects. Quantization and pruning were not considered because pruning risks while removing weights and is crucial for rare medical subtypes, and quantization introduces noise that may harm feature extraction needed for classification. LoRA retains the model’s capability and only adds an adaptive subspace for safe and efficient client-side tuning. Figure 2 shows how each client trains a customized model with a shared base, global adapter, and private classification head. Each FedPerLoRA-Health federated client, that is, a hospital or medical institution, holds private, non-IID blood smear image datasets for leukemia diagnosis.

The local dataset for each client $\dot{i}$ is represented as ${D}_{i}={\left\{\left({x}_{j},{y}_{j}\right)\right\}}_{j=1}^{{n}_{i}}$

where ${x}_{j}^{\left(i\right)}$ is an input image and  ${y}_{j}^{\left(i\right)}$ is the leukemia label. The client uses a composite network of the shared EfficientNet base ${\theta }_{s}$ , the received LoRA adapters ${\theta }_{\text{lora}}^{\left(i\right)}$ , and the local private classification head ${\theta }_{p}^{\left(i\right)}$ to create a personalized model.

### Backbone models: EfficientNet-B0 and B2

All clients in the proposed framework uses the frozen shared base model EfficientNet-B0 or B2. The FedPerLoRA-Health framework instantiates ${\theta }_{s}$ using a frozen EfficientNet architecture that extracts features from all federated clients. Specifically, EfficientNet-B0 and EfficientNet-B2 were adopted as candidate architectures for ${\theta }_{s}$, depending on the computing capability and data complexity. The EfficientNet models use a compound scaling coefficient ${\upphi }_{i}$ to uniformly scale the network depth, width , and input resolution $r$. The scaling relations are defined as $d = \alpha^{\phi } ,\;w = \beta^{\phi } ,\;r = \gamma^{\phi }$ such that $\alpha \cdot \beta^{2} \cdot \gamma^{2} \approx 2$.

The baseline model, EfficientNet-B0, was utilized when $\upphi$ = 0, whereas the scaled-up variant EfficientNet-B2, had greater $w,$ $d$ and resulting in improved accuracy but higher resource utilization. Both variants freeze the EfficientNet model parameters, so ${\theta }_{s}$ remains static during local training and is shared across all clients. The foundation of EfficientNet is the MBConv block, a mobile inverted bottleneck structure with three stages namely expansion layer (1 × 1 convolution), depthwise convolution (3 × 3 or 5 × 5), squeeze-and-excite (SE) module and projection layer (1 × 1). Given an input tensor $x$, the MBConv transformation is as follows:

1 $$"MBConv" (x)=x+"Proj" ("SE" ("Depthwise" ("Expand" (x))))$$

During federated training, the clients do not update the base model ${\theta }_{s}$. Instead, they inserted LoRA adapters inside the blocks 1 × 1 convolutional layers of the MBConv blocks. Each client $i$ optimizes its trainable LoRA adapter weights ${\theta }_{\text{lora}}^{\left(i\right)}$ locally using the client data ${D}_{i}$. B2 is a more expressive feature extractor than B0 (224 × 224) because of its enhanced capacity and resolution (260 × 260 input size). However, this requires more computation. The framework’s versatility allows it to transition between B0 and B2 as ${\theta }_{s}$, depending on the application. Overall, employing EfficientNet-B0 or B2 as the frozen basis ${\theta }_{s}$ and tailored LoRA modules ${\theta }_{\text{lora}}^{\left(i\right)}$ balances the accuracy, communication efficiency and personalization for non-IID leukemia diagnosis.

### Integrating LoRA with EfficientNet

The FedPerLoRA-Health system integrates LoRA modules into the frozen EfficientNet backbone ${\theta }_{s}$ to personalize models and improve communication. Each client $i$ trains a small number of ${\theta }_{\text{lora}}^{\left(i\right)}$ parameters embedded in specific layers of the basic model, instead of fine-tuning the entire set of parameters. In the EfficientNet backbone ${\theta }_{s}$, $W\in {R}^{d\times k}$ is a weight matrix from a $1 \times 1$ convolutional layer in a Mobile Inverted Bottleneck (MBConv) block. This layer was modified by LoRA during training as given below.

2 $$W^{\prime} = W + {\Delta }W,\;{\Delta }W = AB$$

where $A\in {R}^{d\times r}\text{ and }B\in {R}^{r\times k}$ is a trainable low-rank matrix with rank $r\ll \text{min}\left(d,k\right)$ , and $W$ is frozen (part of ${\theta }_{s}$). Thus, each client’s effective parameter set is given by Eq. 3.

3 $${\uptheta }^{\left(i\right)}=\left\{{\uptheta }_{s},{\uptheta }_{\text{lora}}^{\left(i\right)},{\uptheta }_{p}^{\left(i\right)}\right\}$$

The Eq. 4 denotes the personalized forward propagation for client $i$, computed by the improved EfficientNet layer.

4 $${f}_{{\uptheta }_{s}+{\uptheta }_{\text{lora}}^{\left(i\right)}}\left(x\right)={f}_{{\uptheta }_{s}}\left(x\right)+{f}_{{\uptheta }_{\text{lora}}^{\left(i\right)}}\left(x\right)$$

where ${f}_{{\uptheta }_{s}}\left(\cdot \right)$ denotes frozen operations and ${f}_{{\uptheta }_{\text{lora}}^{\left(i\right)}}\left(\cdot \right)$ represents the low-rank modifications of the LoRA modules. The communication overhead is much lower than that of full model updates. The server aggregates these updates using an adaptive optimizer, such as FedAdam and the modified global adapter weights ${\uptheta }_{\text{lora}}^{\left(t+1\right)}$ are redistributed for the following communication round. The framework achieves parameter-efficient fine-tuning while maintaining the generalization ability of the shared EfficientNet backbone by inserting LoRA adapters into selective $1\times 1$ convolutional layer of MBConv blocks. This method allows each client to change the model to its local data distribution without changing the backbone, which is useful in federated learning with non-IID medical image datasets.

### Personalized training at each client

**Fig. 3:**
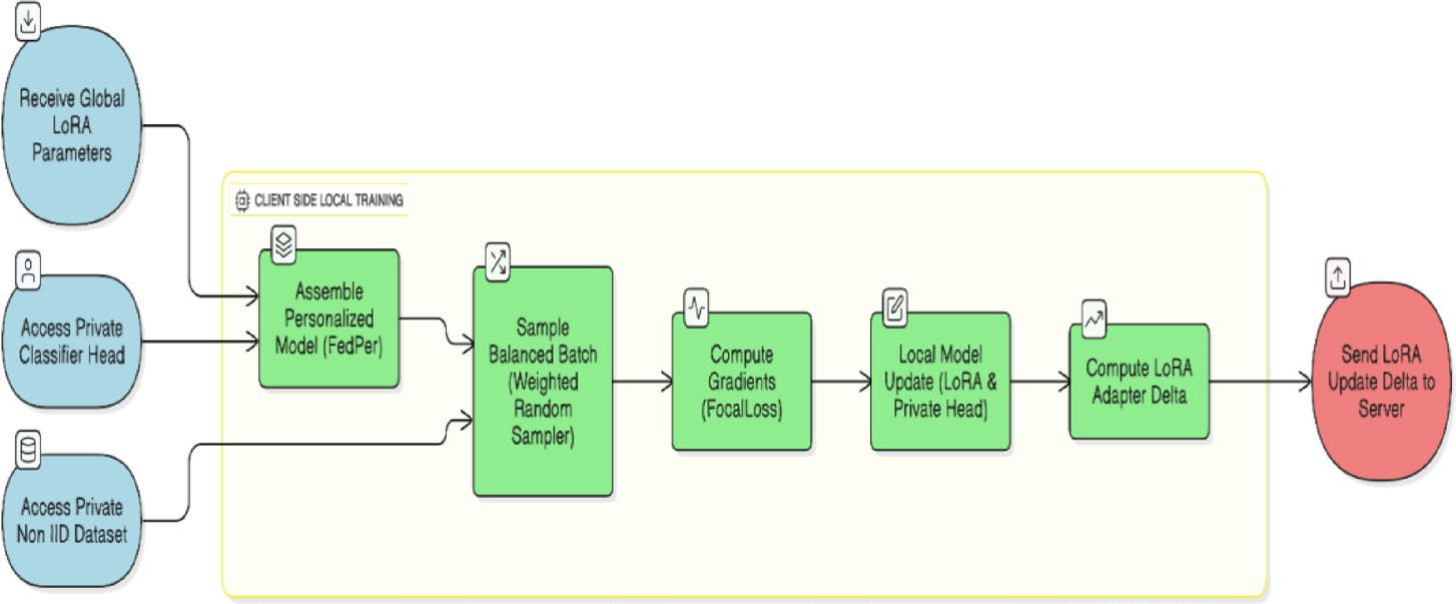
Personalized Federated Learning Process at Client.

As shown in Fig. 3, only the client’s private, non-IID data were used to train this model. A weighted random sampler draws balanced mini-batches during training to reduce class imbalances in medical imaging datasets.

A data sample with labels ${y}_{j}\in \{\text{0,1}\}$ has a sampling probability calculated as given in Eq. 5.

5 $$P\left({y}_{j}\right)=\frac{1}{{f}_{{y}_{j}}}/{\sum }_{k\in \{\text{0,1}\}}\frac{{n}_{k}}{{f}_{k}}$$

where ${f}_{k}$ is the frequency of class $k$ in ${D}_{i}$ and ${n}_{k}$ is the number of samples in class $k$.

To give minority class samples more weight, the probability $P\left({y}_{j}\right)$ is inversely proportional to class frequency. This ensures that local training sufficiently represents the majority and minority class samples without oversampling or data duplication. Class-aware Focal Loss functions down weight simple samples and prioritize hard-to-classify data to optimize the model. This strengthens models in clinical settings with underrepresented disease classes. Each client minimizes a Focal Loss function to handle the class imbalance in the medical dataset, which is given in Eq. 6.

6 $${L}_{\text{focal}}^{\left(i\right)}=-\alpha {\left(1-\widehat{{y}_{j}^{\left(i\right)}}\right)}^{\gamma }\text{log}\left(\widehat{{y}_{j}^{\left(i\right)}}\right)$$

where $\alpha \;\varepsilon \left[ {0,1} \right]$ is the balancing factor for class frequencies, $\upgamma \ge 0$ is the focusing parameter that reduces the loss for well-classified samples and $\widehat{{y}_{j}^{\left(i\right)}}$ is the predicted probability for the true class. The local forward pass for sample ${x}_{j}\in {D}_{i}$ is given in Eq. 7.

7 $$\widehat{{y}_{j}^{\left(i\right)}}={f}_{{\uptheta }_{p}^{\left(i\right)}}\left({f}_{{\uptheta }_{s}+{\uptheta }_{\text{lora}}^{\left(i\right)}}\left({x}_{j}\right)\right)$$

where ${f}_{{\uptheta }_{s}+{\uptheta }_{\text{lora}}^{\left(i\right)}}\left({x}_{j}\right)$ denotes the output feature representation obtained from ${\theta }_{s}$ and ${\theta }_{\text{lora}}^{\left(i\right)}$ and ${f}_{{\uptheta }_{p}^{\left(i\right)}}\left(\cdot \right)$ maps features to the predicted class probability $\widehat{{y}_{j}^{\left(i\right)}}$. For computational efficiency, backpropagation updates only ${\theta }_{\text{lora}}^{\left(i\right)}$ and ${\uptheta }_{p}^{\left(i\right)}$ during local optimization, leaving ${\theta }_{s}$ to be fixed. After local training, the client calculates LoRA delta update as follows:

8 $$\Delta {\uptheta }_{\text{lora}}^{\left(i\right)}={\uptheta }_{\text{lora}}^{\left(i\right)}-{\uptheta }_{\text{lora}}^{\left(t\right)}$$

where the global LoRA status at communication round $t$ is given by ${\theta }_{\text{lora}}^{\left(t\right)}$.

This delta only captures LoRA module alterations, lowering the communication load compared to exchanging full model parameters. The client sends this delta to the central server while maintaining its private data and classification head. This lightweight, tailored training technique optimizes collaboration among distributed medical institutions. FedPerLoRA-Health is ideal for data silos and heterogeneity-prone fields, such as leukemia diagnosis, because it reduces communication overhead as given in Eq. 9 and protects model privacy. Scalable and clinically flexible, the modular framework allows immediate local population customization.

9 $${||\Delta {\theta }_{\text{lora}}^{\left(i\right)}||<<||\theta }_{s}||$$

### Federated training workflow and aggregation

**Fig. 4:**
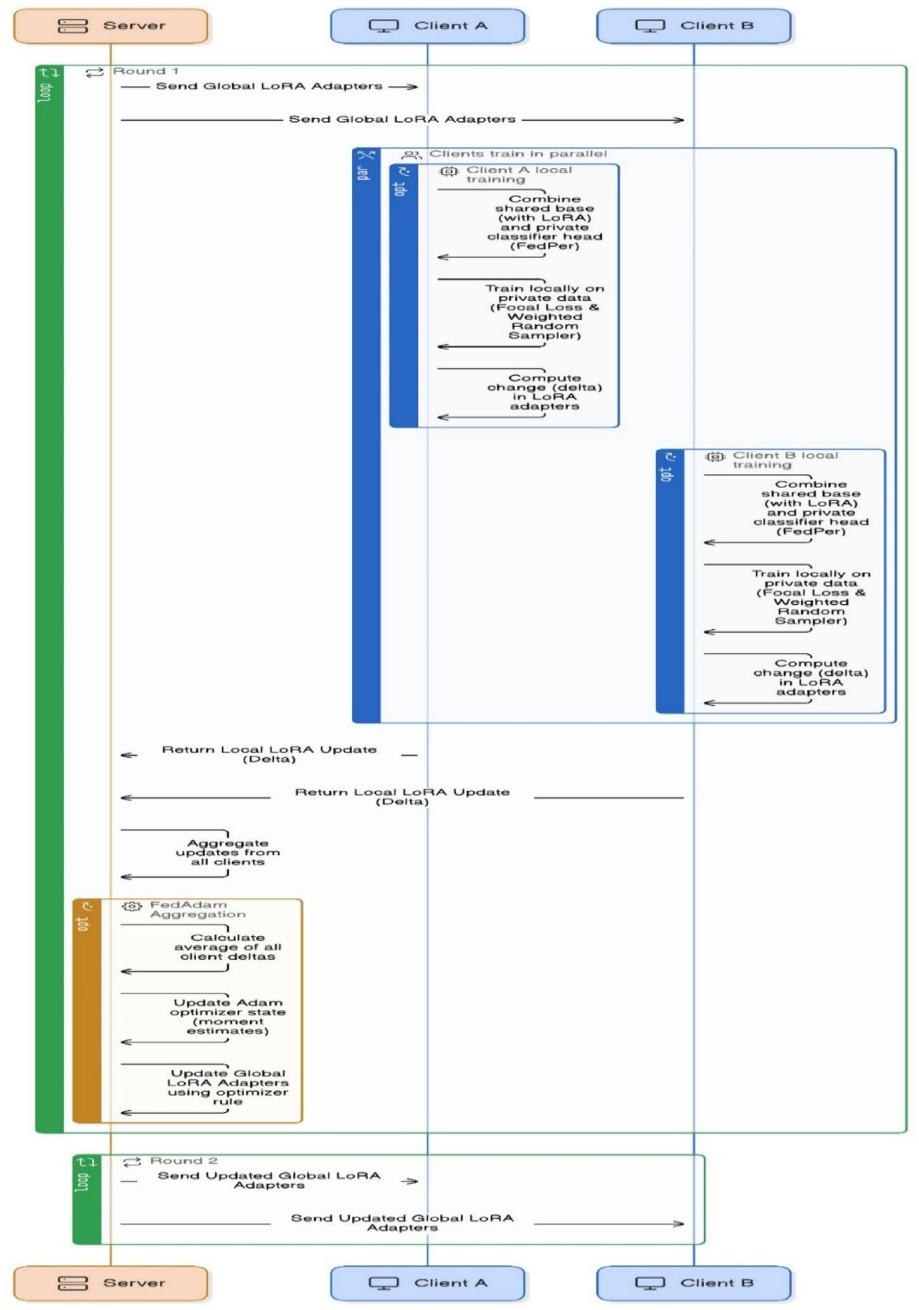
Communication Model between Client and Server.

The workflow of the FedPerLoRA-Health system depicted in Fig. 4 iteratively enables communication-efficient and privacy-preserving leukemia detection across different hospitals. All participating hospitals received global LoRA adapters, lightweight, trainable modules linked to the shared EfficientNet backbone, at the start of each federated learning round. After receiving these adapters, Clients A and B used the FedPer approach to merge the frozen shared base with their private classifier heads and trained their non-IID blood smear datasets locally. The optimization handles class imbalance by using focal loss and weighted random sampling. Each client computes the LoRA adapter parameter delta (update) after training and sends it solely to the server, keeping the raw data and model weights private.

The FedperAdam optimizers with momentum terms stabilize the convergence and combine all LoRA updates on the server. Calculating the average of all client deltas, updating the internal optimizer state variables (moment estimations) and refining the global LoRA adapter parameters using the optimizer rule are all required for redistribution of updated adapters to clients in the next round and continues the loop. This parallelized and lightweight communication loop allows individualized model training while decreasing communication overhead, making it ideal for medical situations with severe data privacy and bandwidth limitations.

**Fig. 5:**
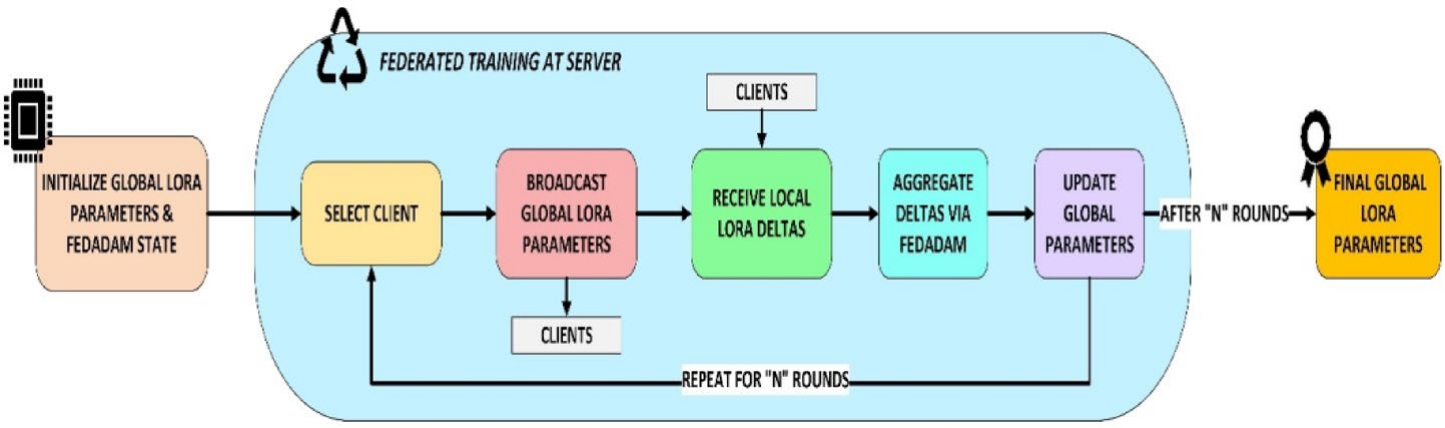
Aggregation of Delta Updates at Server.

In FedPerLoRA-Health, server-side orchestration as shown in Fig. 5, manages communication-efficient and privacy-preserving collaborative training for numerous clients. The global LoRA parameters ${\uptheta }_{\text{lora}}^{\left(0\right)}$ and FedAdam optimizer states for adaptive aggregation were initialized first. The server selects a selection of participating clients in each communication round using a predefined sampling. For local training, the server broadcasts the current global LoRA parameters ${\theta }_{\text{lora}}^{\left(t\right)}$ to all selected clients after client cohort selection. After receiving global parameters, each client fine-tunes its private data and computes the LoRA adapter delta $\Delta {\uptheta }_{\text{lora}}^{\left(i\right)} .$

. Local updates compare the updated and received LoRA weights and are forwarded to the server. After collecting the local deltas from the participating clients, the FedAdam optimizer aggregates them. FedAdam scales updates and stabilizes training using first and second-order moment estimates $\left({m}_{t},{v}_{t}\right)$. Updated global parameters are calculated as follows:

10 $${\uptheta }_{\text{lora}}^{\left(t+1\right)}={\uptheta }_{\text{lora}}^{\left(t\right)}-\upeta \frac{{m}_{t}}{\sqrt{{v}_{t}+\upepsilon }}$$

where $\eta$ represents the learning rate, and $\epsilon$ guarantees numerical stability. The selection, broadcast, local training, delta aggregation and parameter update continue for $N$ communication cycles. The server finalizes the optimal global LoRA settings ${\uptheta }_{\text{lora}}^{\left(N\right)}$ for deployment or customization after these cycles. This federated training strategy preserves data locality and generalizes models across clients for scalable and compliant deployment in privacy-sensitive medical diagnostics. The proposed PerFLR-EffNet Algorithm is given below and reports a complexity as shown in Table 2.

**Table 2:** Complexity Analysis of the Proposed PerFLR-EffNet Algorithm.

Component	Time Complexity (Per Round)	Governed By
Server	$\mathcal{O}\left(\text{K}\cdot {\uptheta }_{\text{LoRA}}\right)$	Number of clients $K ,$ Size of LoRA parameters $\left({\uptheta }_{\text{LoRA}}\right)$
Each Client	$\mathcal{O}\left(E\cdot {D}_{i}\cdot {\uptheta }_{s}\right)$	Local epochs $E ,$ Local data size ${D}_{i}$ , Frozen backbone size ${\uptheta }_{s}$
Overall	$\mathcal{O}\left(T\cdot K\cdot E\cdot {D}_{i}\cdot {\uptheta }_{s}\right)$	All above factors, plus number of rounds $T$

**Algorithm 1:**
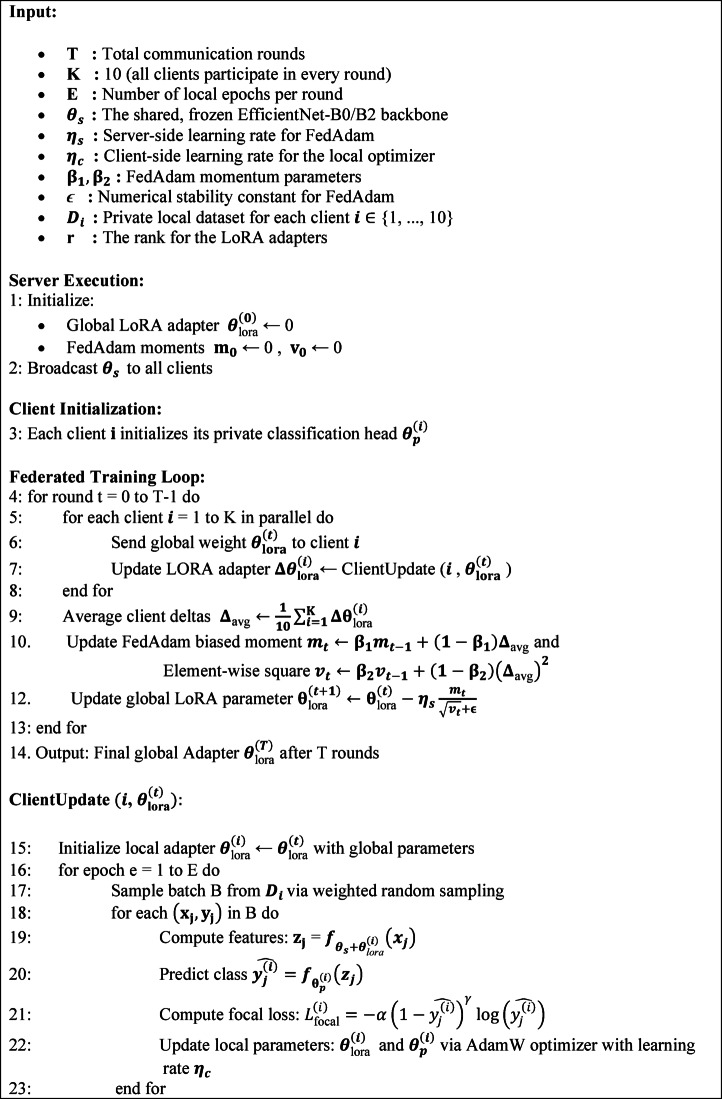
Proposed PerFLR-EffNet Algorithm.

The complexity of the proposed PerFLR-EffNet algorithm, including the server, client and overall training round time costs was analyzed. The server-side complexity is related to the number of clients ($K$) and the size of the LoRA parameters (${\uptheta }_{\text{LoRA}}$), indicating a small aggregation overhead $\mathcal{O}\left(\text{K}\cdot {\uptheta }_{\text{LoRA}}\right)$. The cost for each client is determined to be $\mathcal{O}\left(E\cdot {D}_{i}\cdot {\uptheta }_{s}\right)$ given by the number of local epochs ($E$), local dataset size (${D}_{i}$) and frozen backbone size (${\uptheta }_{s}$), which accounts for most of the computational work during training. The final complexity is $\mathcal{O}\left(T\cdot K\cdot E\cdot {D}_{i}\cdot {\uptheta }_{s}\right)$ by combining the contributions from all rounds ($T$). LoRA’s parameter-efficient architecture keeps server costs low, whereas clients shoulder most of the computing load.

## Experimental setup

### Dataset description

This research used the publicly available "Blood Cells Cancer (ALL) dataset" of microscopic blood smear images curated for ALL identification. The collection consists of 3242 colour images that represent the leukemia cell development phases and subtypes as Benign, Early Pre-B, Pre-B and Pro-B. Expert annotation labels each image to ensure supervised learning reliability. In the dataset, staining, cell shape and background noise varies, mimicking non-IID conditions found in real-world medical data from different hospitals. This makes it a good baseline for federated and personalized learning systems like FedPerLoRA-Health. The collection is split between synthetic clients representing hospital facilities to imitate the federated environments. Subsets of data with class imbalance and domain shifts are sent to each client, duplicating localized population distributions and diagnostic variability. Under communication-efficient, parameter-restricted LoRA modules and federated personalization algorithms, this segmentation allows for client-specific personalization performance measurement. All raw blood smear images are scaled to $224 \times 224$ pixels for uniformity and interoperability with the EfficientNet based study backbone as discussed in section "Architecture and process flow of the FedPerLoRA-health framework". The channel-wise mean and standard deviation from the dataset were used to normalize the images to meet the EfficientNet pretraining settings. The dataset is configured as illustrated in Table 3 as follows.

**Table 3:** Dataset Configuration.

Parameter	Value
Total Images	3242
Data Distribution	Non-IID
Training Split	72%
Validation Split	8%
Test Split	20%
Training Samples	2328
Validation Samples	265
Test Samples	649

For the personalized federated learning, the client-wise data distribution is listed in Table 4 as shown below.

**Table 4:** Client-wise Data Distribution.

Client	Dataset	Total	Benign (%)	Pre-B (%)	Pro-B (%)	Early Pre-B (%)
Client 0	Train	240	41 (17.1)	50 (20.8)	73 (30.4)	76 (31.7)
	Validation	27	4 (14.8)	6 (22.2)	8 (29.6)	9 (33.3)
Client 1	Train	212	34 (16.0)	79 (37.3)	38 (17.9)	61 (28.8)
	Validation	24	4 (16.7)	9 (37.5)	4 (16.7)	7 (29.2)
Client 2	Train	234	32 (13.7)	111 (47.4)	36 (15.4)	55 (23.5)
	Validation	27	4 (14.8)	13 (48.1)	4 (14.8)	6 (22.2)
Client 3	Train	243	32 (13.2)	67 (27.6)	60 (24.7)	84 (34.6)
	Validation	28	4 (14.3)	7 (25.0)	7 (25.0)	10 (35.7)
Client 4	Train	282	56 (19.9)	59 (20.9)	72 (25.5)	95 (33.7)
	Validation	32	6 (18.8)	7 (21.9)	8 (25.0)	11 (34.4)
Client 5	Train	270	45 (16.7)	74 (27.4)	59 (21.9)	92 (34.1)
	Validation	31	5 (16.1)	9 (29.0)	7 (22.6)	10 (32.3)
Client 6	Train	195	30 (15.4)	58 (29.7)	55 (28.2)	52 (26.7)
	Validation	22	3 (13.6)	7 (31.8)	6 (27.3)	6 (27.3)
Client 7	Train	223	42 (18.8)	66 (29.6)	52 (23.3)	63 (28.3)
	Validation	25	5 (20.0)	7 (28.0)	6 (24.0)	7 (28.0)
Client 8	Train	216	38 (17.6)	71 (32.9)	48 (22.2)	59 (27.3)
	Validation	25	4 (16.0)	8 (32.0)	6 (24.0)	7 (28.0)
Client 9	Train	213	18 (8.5)	50 (23.5)	79 (37.1)	66 (31.0)
	Validation	24	2 (8.3)	6 (25.0)	9 (37.5)	7 (29.2)

The hyper-parameters are configured as shown below in Table 5 in order to achieve better accuracy and communication efficiency. The hyperparameters were empirically evaluated on a validation set and follow federated learning and parameter-efficient fine-tuning literature. The Dirichlet concentration parameter (α = 0.7) creates genuine heterogeneity, while a single local epoch (E = 1) reduces client drift. The LoRA rank (r = 16) optimizes adaptation and parameter efficiency. The focal Loss settings (γ = 2.0, α = 0.25) were selected to solve class imbalance and defined learning rates for steady convergence in server aggregation and client-side adaptation.

**Table 5:** Hyper-parameters of PerFLR-EffNet.

Category	Parameter	Value
Federated Learning	Aggregation Method	FedAdam
	Communication Rounds	20
	Number of Clients	10
	Local Epochs per Round	1
	Data Distribution (α)	Non-IID (Dirichlet, α = 0.7)
LoRA	Rank (r)	16
	Alpha	32
	Dropout	0.1
	Target Modules	Conv2d layers with groups = 1
Training & Optimization	Data Sampler	WeightedRandomSampler
	Loss Function	Focal Loss (γ = 2.0, α = 0.25)
	Optimizer	Adam
	Client Learning Rate	1e^-4^
	Server Learning Rate	0.01
	Batch Size	32

### Evaluation metrics

Traditional classification metrics and Federated Learning (FL)-specific efficiency indicators are used to evaluate the FedPerLoRA-Health system. Leukemia detection accuracy and robustness across varied client datasets are measured using classification metrics, whereas FL-specific metrics assess federated optimization’s communication overhead and convergence behavior.

- Accuracy: The percentage of accurately categorized leukemia cell images indicates prediction accuracy.
  11 $${\text{Accuracy}}=\frac{\text{TP}+\text{TN}}{\text{TP}+\text{TN}+\text{FP}+\text{FN}}$$
- Precision – Recall Curve: This metric shows the trade-off between Precision (accurate positive predictions) and Recall (complete positive predictions). Blood smear images are uneven; therefore, the PR curve area is useful.
  12 $${\text{Precision}} = \frac{TP}{{TP + FP}}$$
  13 $${\text{Recall}}=\frac{TP}{TP+FN}$$
- ROC Curve: Compares True Positive Rate (TPR) against False Positive Rate (FPR) across thresholds. The classifier’s AUC summarizes its class distinction abilities.
  14 $${\text{TPR}}=\frac{TP}{TP+FN}$$
  15 $${\text{FPR}}=\frac{FP}{FP+TN}$$
- F1-Score: Harmonic mean of precision and recall balances both metrics.
  16 $$F1\text{-score}=2\cdot \frac{{\text{Precision}}\cdot {\text{Recall}}}{{\text{Precision}}+{\text{Recall}}}$$
- Cohen’s Kappa (κ): This is a measure of agreement between anticipated and actual labels, considering the chance. Note that ${p}_{0}$ represents observed accuracy, ${p}_{e}$ represents the anticipated agreement in Eq. 17.
  17 $$\kappa =\frac{{p}_{o}-{p}_{e}}{1-{p}_{e}}$$

MCC : This is a balanced measure of binary classification quality, particularly in cases of class imbalance as given in Eq. 18.

18 $$MCC=\frac{TP\cdot TN-FP\cdot FN}{\sqrt{\left(TP+FP\right)\left(TP+FN\right)\left(TN+FP\right)\left(TN+FN\right)}}$$

Metrics for federated learning

Metrics to assess FedPerLoRA-Health’s federated efficiency and scalability such as Communication cost and convergence rate are used.

- Cost per Round of Communication: Quantifies the server-client data transmitted each communication round (MB) as given in Eq. 19. The communication footprint is much lower than full-model updates because only LoRA adapters and classification heads are sent.
  19 $$\text{Cost per Round}=2\times \text{Model Size (MB)}\times \text{Clients per Round}$$
- Rounds to Convergence: Denotes the number of communication rounds needed for the global model to reach a convergence criterion (e.g., target accuracy or loss) as given in Eq. 20. Fewer rounds indicate better communication in learning.
  20 $$\text{Rounds to Convergence}=\text{min}\{r:{L}_{r}\le {L}_{\text{threshold}}\text{ or }{\text{Accuracy}}_{r}\ge {\text{target}}\}$$

## Results and discussion

**Fig. 6:**
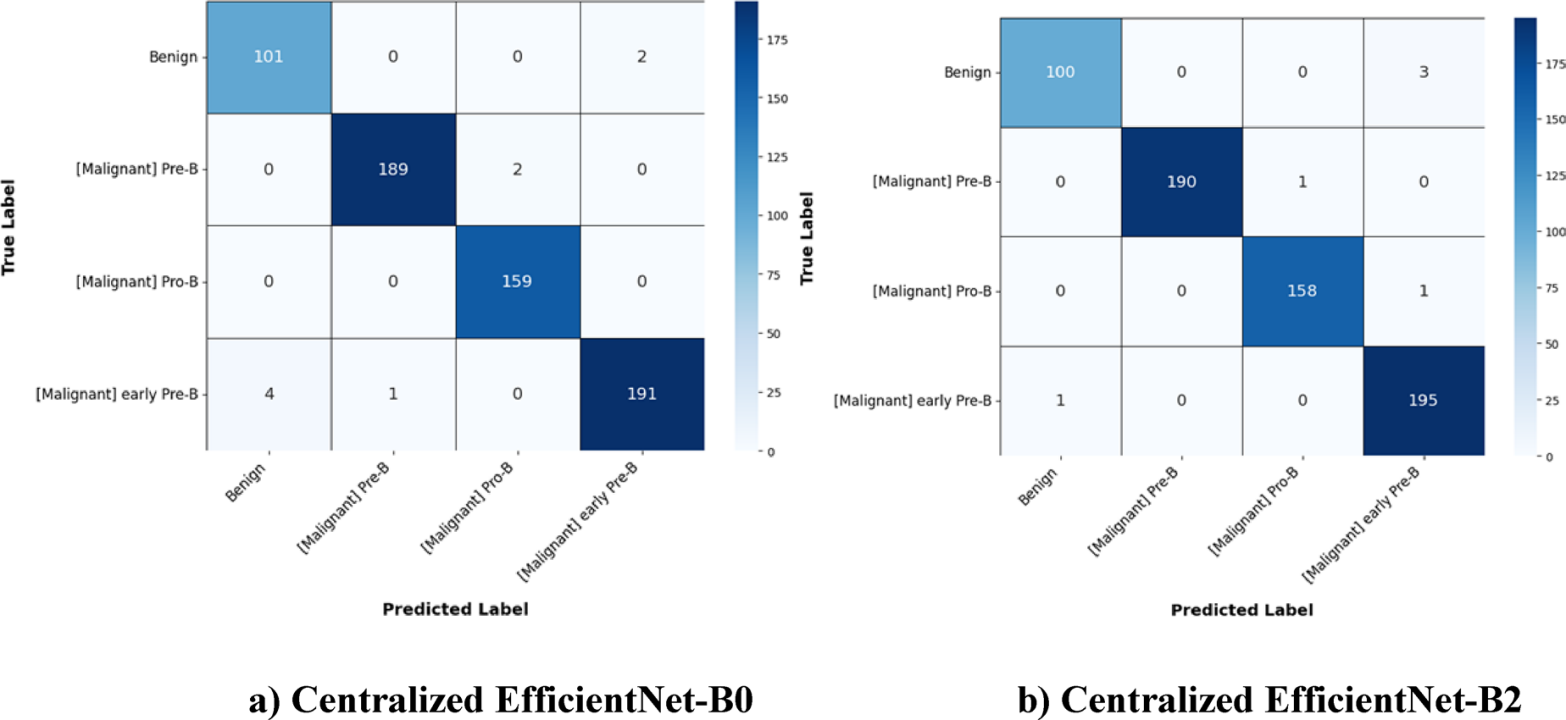
Confusion Matrix of Centralized Models.

**Fig. 7:**
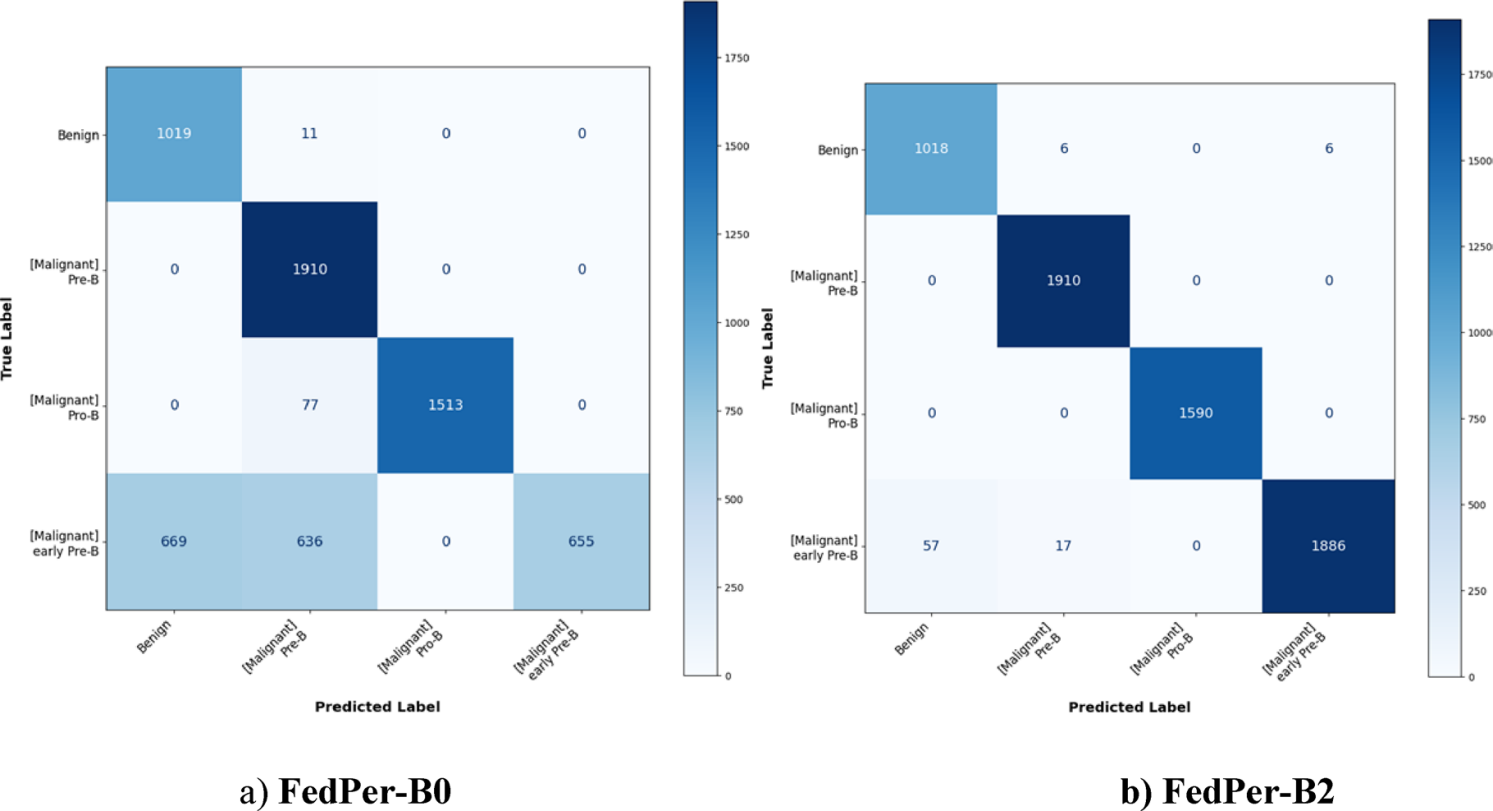
Confusion Matrix of Personalized Federated Models.

The proposed FedPerLoRA-Health framework is analysed with two variants of EfficientNet namely EfficientNet-B0 and EfficientNet-B2. These models were applied in a centralized manner and also in personalized federation among the clients as FedPer-B0 and FedPer-B2. The performance of these variants was analysed based on the metrics listed in “Evaluation metrics”. The confusion matrix of the centralized EfficientNet-B0, EffecientNet-B2 are shown in Fig. 6 and the aggregated confusion matrix of personalized federated models FedPer-B0 and FedPer-B2 are shown in Fig. 7.

To assess the centralized model’s classification performance, confusion matrices for EfficientNet-B0 and B2 were examined. Both models attempt to discriminate benign, Pre-B (Malignant), Pro-B (Malignant) and early Pre-B (Malignant). Both models have good classification accuracy, but EfficientNet-B2 reduces false positives in crucial malignant categories better. This suggests it could aid early and accurate leukemia subtype identification in centralized diagnostic systems.

The confusion matrices in Fig. 7 show the effectiveness of FedPerLoRA-Health with deeper backbones and personalization layers. The FedPer-B0 performs well for Pre-B and Pro-B, but early Pre-B confuses it with Benign and Pre-B, suggesting a class imbalance or overlapping feature representations. FedPer-B2 improves upon FedPer-B0, especially in distinguishing the early Pre-B class. Owing to its stronger backbone, EfficientNet-B2 improves feature representation and generalization, reducing misclassifications across all classes. FedPer-B0 struggles with class separation for early Pre-B leukemic cells, while FedPer-B2 dramatically reduces these mistakes, showing that more expressive models such as EfficientNet-B2, better capture small morphological changes across ALL kinds.

The two centralized models were compared to provide a performance benchmark. Given their full training dataset access, the EfficientNet-B0 and B2 architectures perform well. All measures as depicted in Table 6 show a slight but continuous advantage for the EfficientNet-B2 model. The F1-Score, Matthews Correlation Coefficient (MCC), and Cohen’s Kappa score increased from 0.9812 to 0.9903 as EfficientNet-B2 enhanced test accuracy from 98.31 to 99.38%. The incremental performance increase shows that EfficientNet-B2’s architectural capacity allows it to capture more discriminative features even when trained on a large dataset. Thus, the proposed federated learning frameworks are evaluated against the more rigorous gold-standard baseline, the centralized EfficientNet-B2.

**Table 6:** Performance of Centralized Models (EfficientNet-B0 vs. EfficientNet-B2).

Metric	EfficientNet-B0	EfficientNet-B2
Final Test Accuracy	0.9831	0.9938
Final Test F1-Score	0.9830	0.9938
Matthews Correlation Coefficient (MCC)	0.9812	0.9903
Cohen’s Kappa Score	0.9812	0.9896

A critical analysis of the FedPer-B0 and FedPer-B2 federated models depicted in Table 7 shows that the backbone architecture’s representational capabilities is crucial for success in a non-IID context. While functional, the FedPer-B0 architecture has a moderate average accuracy of 78.54% and severe performance volatility, with Matthews Correlation Coefficient (MCC) values ranging from 0.065 on Client 5 to 0.759 on Client 6. It cannot generalize across statistically diverse data silos. In contrast, FedPer-B2 has a state-of-the-art average accuracy of 98.67%. More importantly, it shows remarkable robustness, improving performance for all clients and narrowing the performance gap. For example, Client 5’s MCC almost six-folded to 0.405, while Client 2’s reached 0.874. EfficientNet-B2’s increased architectural complexity is not only beneficial but essential, allowing it to learn discriminative features that are generalizable across diverse client data distributions and overcome the main challenge of federated learning. FedPer-B2 exceeds FedPer-B0 for every client in terms of accuracy, F1-score, Kappa, and MCC. The improvement is significant, with the average accuracy increasing from 0.7854 to 0.9867 and the F1-Score from 0.7560 to 0.9868. This shows that FedPer-B2 customization is more consistent and reliable across clients and models.

**Table 7:** Performance of Personalized Federated Models (FedPer-B0 vs. FedPer-B2).

Client	FedPer-B0				FedPer-B2			
	Accuracy	F1-Score	Kappa	MCC	Accuracy	F1-Score	Kappa	MCC
Client 0	0.7812	0.7498	0.3584	0.3663	0.9861	0.9862	0.5925	0.5944
Client 1	0.7643	0.7223	0.2683	0.2846	0.9877	0.9877	0.6669	0.6781
Client 2	0.7797	0.7561	0.1889	0.1968	0.9892	0.9892	0.8706	0.8739
Client 3	0.7720	0.7385	0.4208	0.4300	0.9892	0.9892	0.6260	0.6488
Client 4	0.7720	0.7344	0.6533	0.6603	0.9861	0.9861	0.7158	0.7297
Client 5	0.7843	0.7517	0.0578	0.0651	0.9846	0.9846	0.3950	0.4048
Client 6	0.8105	0.7914	0.7549	0.7590	0.9877	0.9877	0.8459	0.8466
Client 7	0.7904	0.7664	0.3158	0.3543	0.9846	0.9846	0.7257	0.7354
Client 8	0.7874	0.7591	0.6257	0.6322	0.9861	0.9861	0.7405	0.7425
Client 9	0.8120	0.7908	0.3506	0.3820	0.9861	0.9862	0.7475	0.7496
Average	0.7854	0.7560	0.3997	0.4139	**0.9867**	**0.9868**	**0.6926**	**0.7004**

 Table 8 shows that the personalized federated models performed better after switching from FedPer-B0 to FedPer-B2. FedPer-B0 had a mean accuracy of 0.7854, whereas FedPer-B2 greatly exceeded it with 0.9867, supported by tight confidence intervals and a significant *p*-value (< 0.001). FedPer-B0 had an F1 score of 0.7560, and FedPer-B2 0.9868, with narrow intervals and great statistical significance. These findings demonstrate that FedPer-B2 is suitable for robust medical diagnostic applications because it improves the reliability and effectiveness of personalized federated learning. FedPer-B2 showed significant improvements in Accuracy and F1-Score, as proven by *p* < 0.001. Accuracy [0.9856, 0.9878] had minimal variability and good model confidence. This shows FedPer-B2’s superior and statistically dependable performance over FedPer-B0.

**Table 8:** Evaluation of FedPer Models with Confidence Intervals and Statistical Significance.

Metric	Model	Mean	Standard Deviation (+ /-)	Confidence Interval (95%)	*p*-value
Accuracy	FedPer-B0	0.7854	0.0151	[0.7750,0.7958]	–
	FedPer-B2	**0.9867**	**0.0017**	**[0.9856,0.9878]**	** < 0.001**
F1 Score	FedPer-B0	0.7560	0.0225	[0.7405,0.7715]	–
	FedPer-B2	**0.9868**	**0.0016**	**[0.9858,0.9878]**	** < 0.001**

**Fig. 8:**
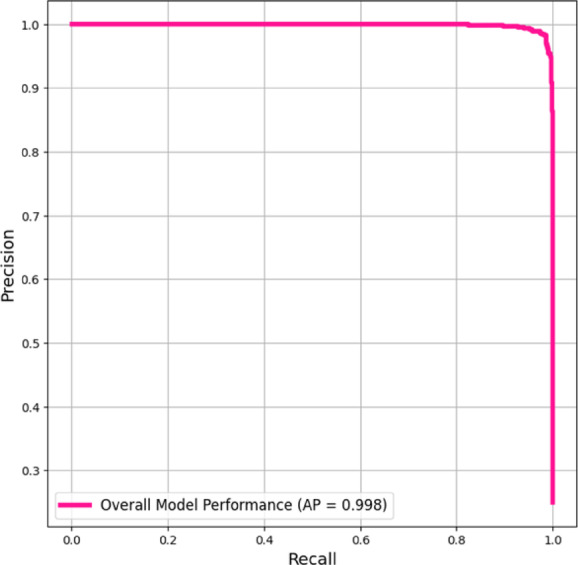
Precision-Recall Curve (Micro-averaged) of EfficentNet-B0.

The precision-recall curve in Fig. 8 shows that the centralized EfficientNet-B0 model performed well, with a near-perfect Average Precision (AP) score of 0.998. The model consistently had high precision across all recall levels (0.0–1.0), demonstrating its ability to properly identify positive cases with low false positives. The model’s excellent precision values, even at full recall, indicate wide coverage and dependable predictions, making it a promising categorization solution. These results demonstrate the model’s excellent discriminative capacity when trained on centralized data, making it an appropriate benchmark for federated learning comparisons.

**Fig. 9:**
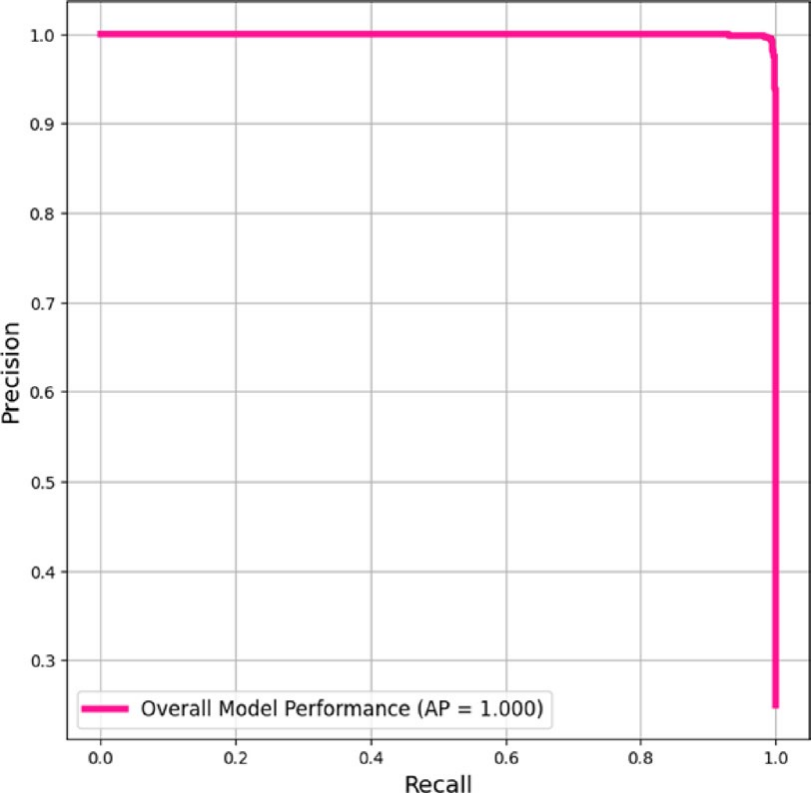
Precision-Recall Curve (Micro-averaged) of EfficentNet-B2.

A theoretically flawless AP of 1.000 for the centralized EfficientNet-B2 model as shown in Fig. 9 indicates an optimum precision-recall balance across all classification thresholds. The model had maximal precision (100% positive predictive value) for all recall (sensitivity) levels from 0.0 to 1.0. The upper-bound capacity of the architecture, not the real-world performance, should be interpreted from this optimal performance under controlled evaluation settings.

**Fig. 10:**
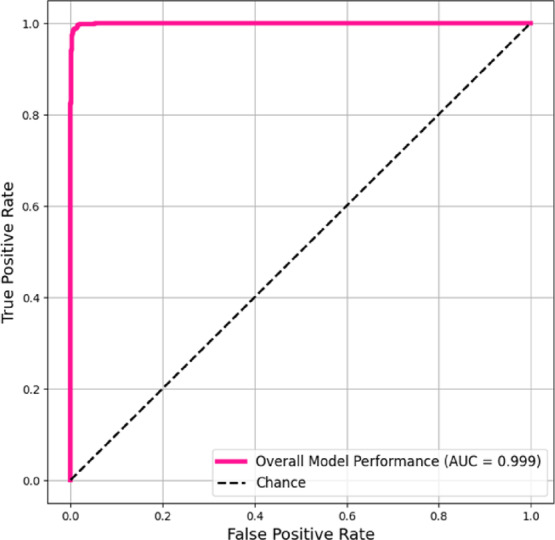
ROC – AUC Curve (Micro-averaged) of EfficentNet-B0.

**Fig. 11:**
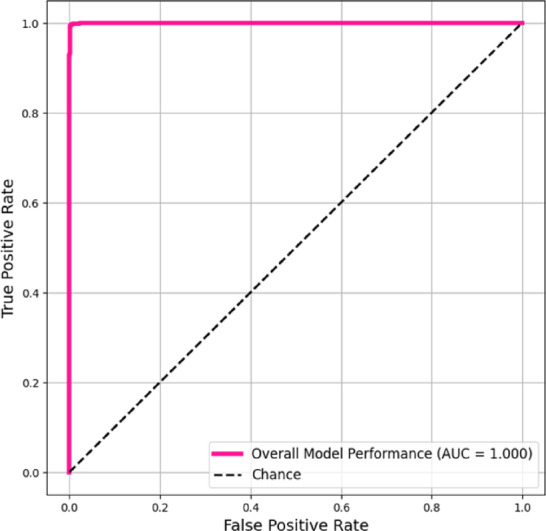
ROC – AUC Curve (Micro-averaged) of EfficentNet-B2.

An AUC of 0.999 indicates excellent classification performance for the EfficientNet-B0 model as shown in Fig. 10. This result shows the near-flawless discrimination of the model, with its ROC curve approaching the ideal top-left corner. The model’s 0.999 AUC exceeded chance-level classification (AUC = 0.5), proving its ability to discriminate between positive and negative cases across all decision thresholds. The model’s high sensitivity and specificity make it reliable for clinical decision support. When trained on extensive centralized data, EfficientNet-B0 was highly effective for this diagnostic task. With an AUC of 1.000 in the test set as in Fig. 11, the EfficientNet-B2 model showed theoretically ideal discrimination, suggesting flawless class separation under the given assessment conditions.

**Fig. 12:**
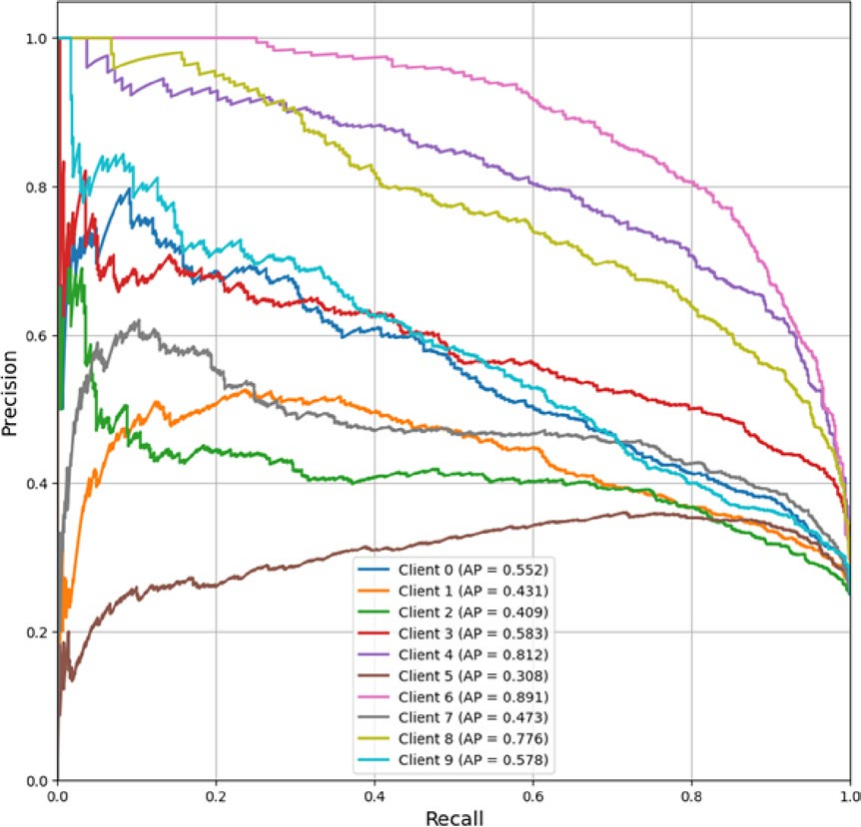
Precision-Recall curve (Micro-averaged) of FedPer-B0.

The FedPer-B0 model’s Precision-Recall curves as shown in Fig. 12 vary widely by client, with AP scores ranging from 0.308 (Client 5) to 0.891 (Client 6). Some clients had high precision (AP > 0.77), others clustered near-randomly, and most clustered moderately. This mismatch may be related to architectural restrictions because the model cannot generalize robustly across varied data distributions. Owing to its dependence on local data, FedPer-B0 is unreliable for uniform rollout.

**Fig. 13:**
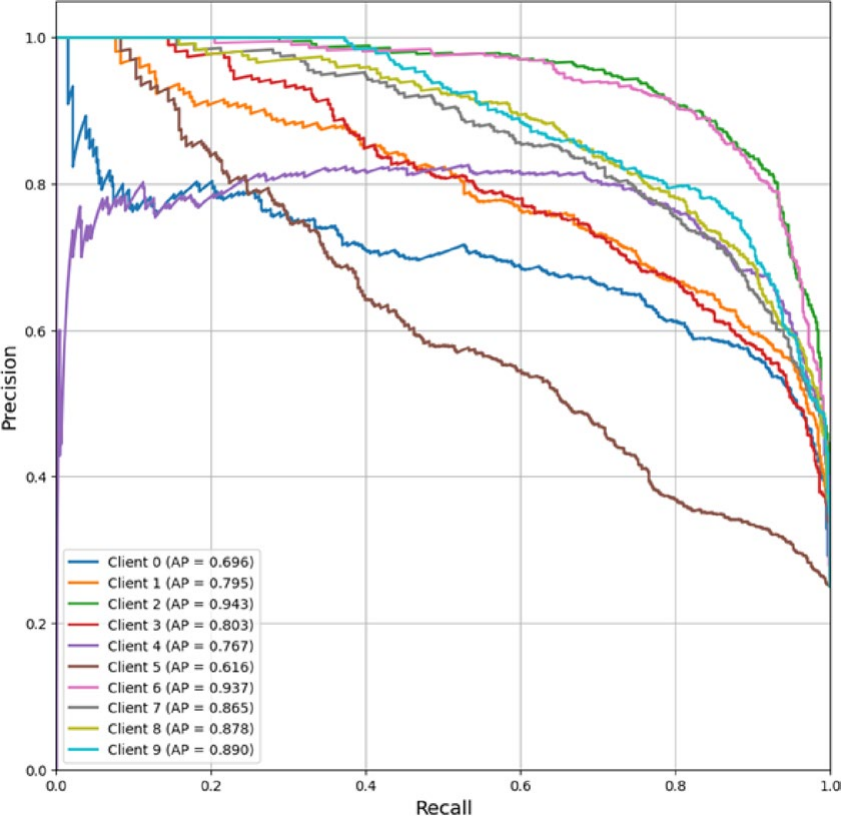
Precision-Recall curve (Micro-averaged) of FedPer-B2.

PR curves of FedPer-B2 as in Fig. 13 show good and consistent performance across customers, with AP scores mostly in the high range (0.767–0.943). Clients 2 (0.943), 6 (0.937), and 9 (0.890) had excellent AP values (> 0.8). Only Client 5 had a somewhat lower AP (0.616), indicating a weakness in its data distribution. The close clustering of high AP values suggests that FedPer-B2 has robust predictive dependability across various customers, with most precision values consistent even at higher recall levels (0.6–1.0). The model’s ability to acquire generalizable features in federated settings and handle non-IID data distributions explains its excellent persistent performance.

**Fig. 14:**
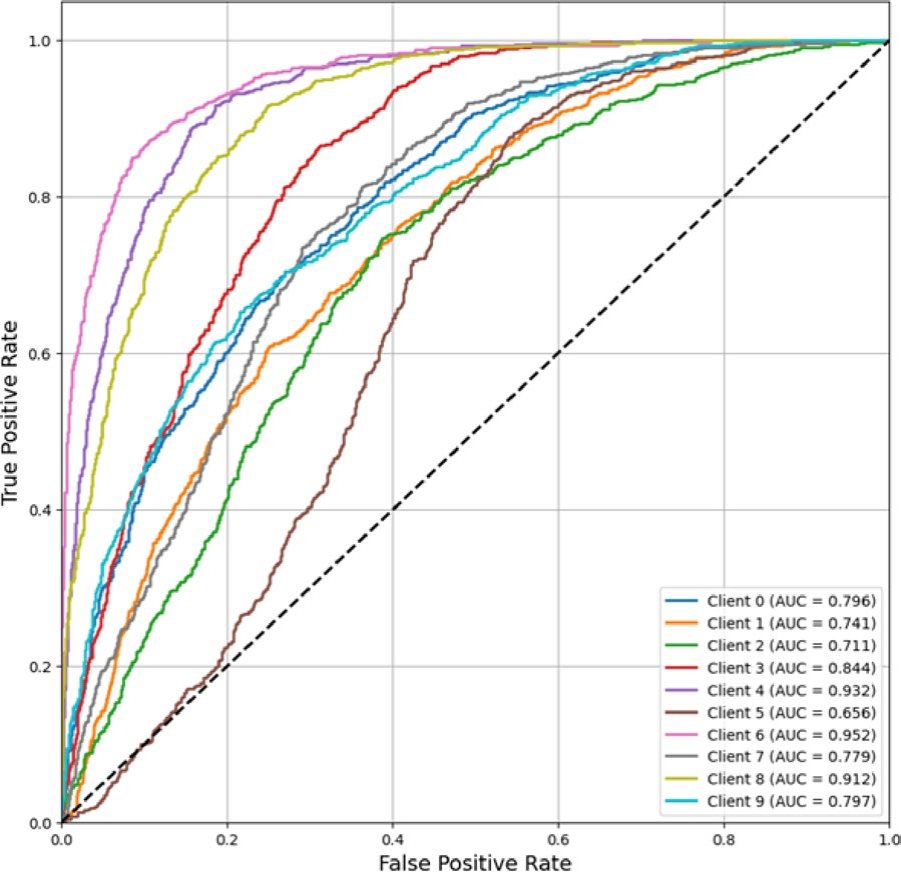
ROC-AUC curve (Micro-averaged) of FedPer-B0.

The FedPer-B0 model showed moderate to strong discrimination among clients as shown in Fig. 14, with AUC scores ranging from 0.656 (Client 5) to 0.952 (Client 6). Most clients (7/10) achieved an AUC > 0.75, indicating good classification performance. However, there was significant variation, with three (4, 6, 8) achieving excellent performance (AUC > 0.9), two (1, 5) marginally performing (AUC < 0.75), and the rest demonstrating solid mid-range performance (0.75–0.85 This shows that FedPer-B0 can perform well for some clients but inconsistently across data distributions, possibly due to the model’s limited ability to adapt to all non-IID data features in the federated context. High-performing (Client 6:0.952) and low-performing (Client 5:0.656) cases demonstrate the difficulty in maintaining uniform effectiveness with this design in heterogeneous federated contexts.

**Fig. 15:**
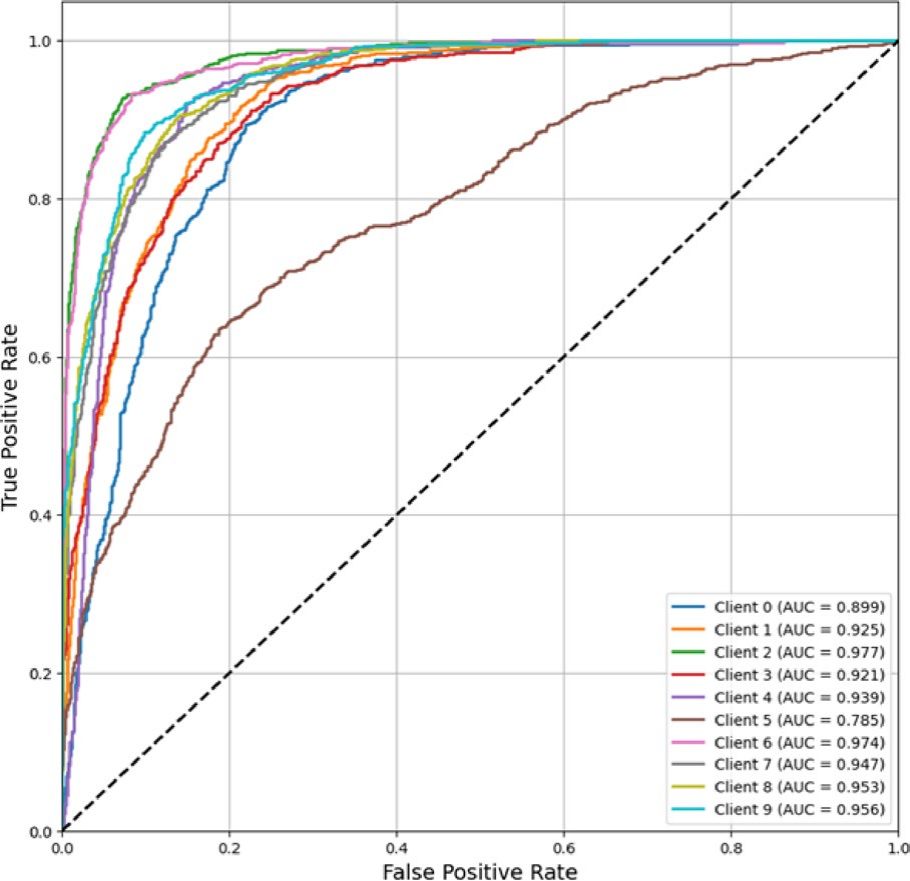
ROC-AUC curve (Micro-averaged) of FedPer-B2.

In Fig. 15, FedPer-B2’s ROC-AUC curves is depicted. At AUC scores of 0.785–0.977, the FedPer-B2 model discriminated well across all the clients. Nine of ten clients performed over 0.9 AUC, with Clients 2 (0.977), 6 (0.974), and 9 (0.956) performing well. Client 5 exhibited clinically relevant discrimination despite a lower AUC (0.785). FedPer-B2 learns robust and generalizable features while protecting data privacy in federated contexts, as shown by its good AUC scores across institutions. The model handles varied data distributions well, with most customers achieving near-perfect classification performance (AUC > 0.95).

The federated learning-based efficiency analysis of the proposed FedPer-B0 and FedPer-B2 are analysed with the metrics as given in section "Evaluation metrics" shown in Tables 9 and 10. As aligned with FedPer, there are several approaches such as FedProx, pFedMe and FedPer ++ . FedProx restricts local updates to handle statistical heterogeneity but does not allow client-specific customization, thereby limiting its use in non-IID medical data contexts. pFedMe provides meta-learning-inspired regularization for personalization; however, its high computational overhead and slower convergence due to several local updates every round make it unsuitable for large-scale or resource-constrained healthcare situations. FedPer ++ adds personalization tactics but increases communication and storage requirements, which may be restricted in federated medical implementations with limited bandwidth and device capabilities. Owing to its communication efficiency, FedPer was used in the proposed work.

**Table 9:** Communication Efficiency of the Proposed FedPer-B0.

Metric	EfficientNet-B0	Proposed FedPer-B0	Reduction (%)
Trainable Parameters	5,288,548	664,368	87.44
Communication Cost (Per Round)	20.17 MB	2.53 MB	87.44

**Table 10:** Communication Efficiency of the Proposed FedPer-B2.

Metric	EfficientNet-B2	Proposed FedPer-B2	Reduction (%)
Trainable Parameters	9,109,994	1,079,024	88.16
Communication Cost (Per Round)	34.75 MB	4.12 MB	88.16

The injection of LoRA into the proposed federated framework notably improves communication efficiency, which is essential for federated learning in resource-constrained contexts. Table 9 shows how FedPer-B0 reduces the number of trainable parameters from 5,288,548 in the entire EfficientNet-B0 model to 664,368. A significant 87.44% decrease was observed, and the communication overhead decreased proportionally with the parameter efficiency. Lightweight 2.53 MB LoRA adapters were sent instead of the entire 20.17 MB model in each round of federated learning. This 87.44% reduction in transmission cost per round reduces the network bandwidth and energy usage for participating clients, making the proposed federated learning system more scalable and feasible.

Table 10 shows that the proposed FedPer-B2 reduces the computational and transmission overheads much further than the EfficientNet-B2 backbone. The FedPer-B2 model has 1,079,024 trainable parameters, which is 88.16% less than the centralized EfficientNet-B2 model. Communication expenses reflect this efficiency in the study. The data payload is substantially smaller, from 34.75 to 4.12 MB every round. This 88.16% decrease in communication burden is crucial and this proves that the FedPer framework allows clients with low network bandwidth to use advanced and high-performance models.

**Fig. 16:**
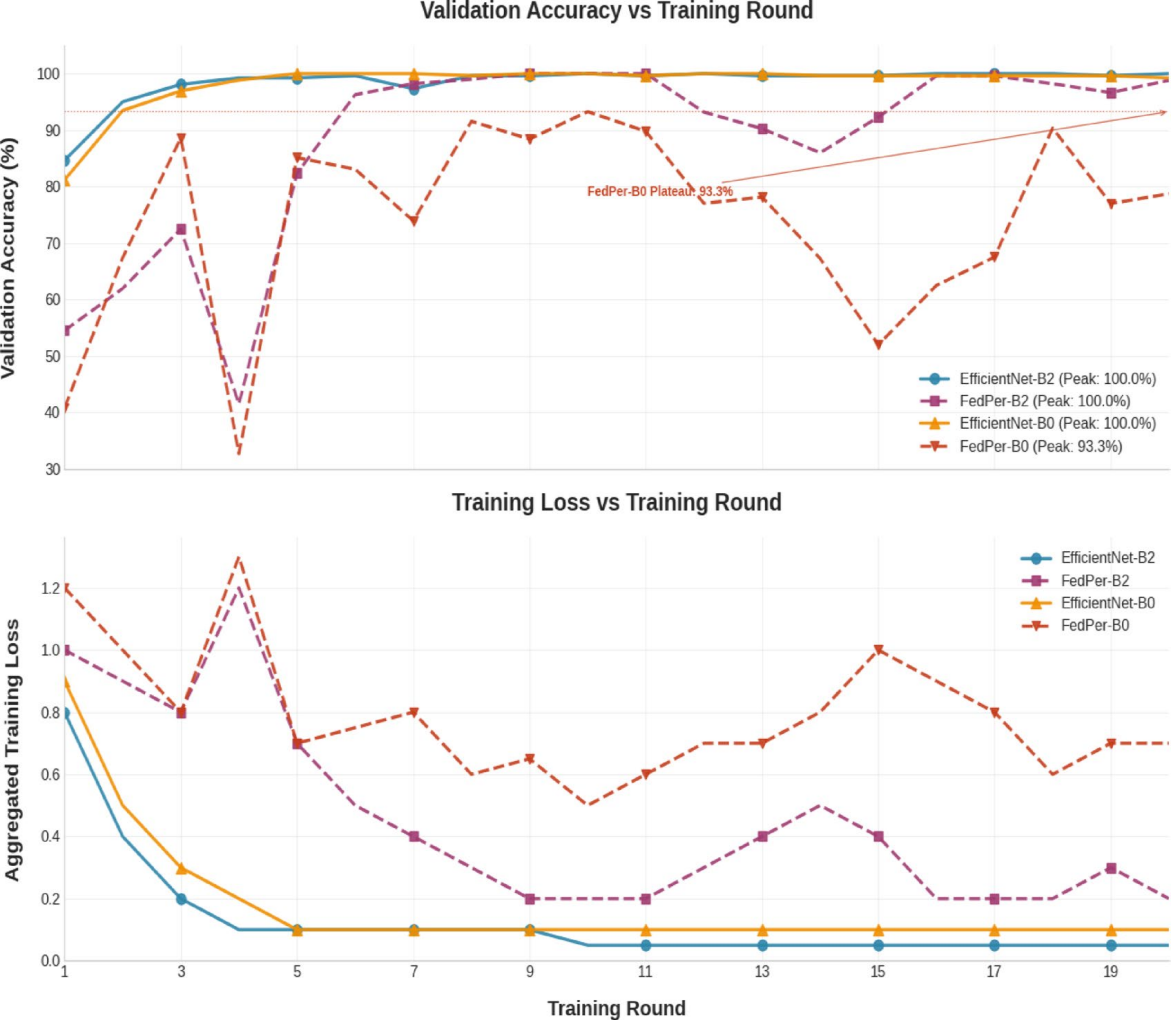
Convergence Rate.

The convergence profile of these strategies was depicted in Fig. 16. Different architectures and training methodologies exhibited different convergence patterns in the validation accuracy rates. Both centralized models EfficientNet-B0, B2 and FedPer-B2 fit the validation data with 100% correctness, whereas FedPer-B0 peaked at 93.26%, showing model capacity limitations in processing non-IID data distributions. FedPer-B2 equals the peak performance of its centralized counterpart, demonstrating that it is more adaptable to federated settings than FedPer-B0. The unquantified training loss curves should exhibit faster convergence for B2-based models than for B0 variants, a larger final loss for FedPer-B0, and stable optimization for FedPer-B2, mimicking centralized training dynamics. These findings show that architectural capacity is crucial for federated learning stability, as larger models (B2) better adjust for data heterogeneity and retain convergence qualities, such as centralized training.

In order to highlight the contribution of each component of this work and to examine how the model components affected the performance and communication efficiency, an ablation study was conducted and the results were shown in Table 11. The base EfficientNet-B0/B2 models lack federated or personalized components. FedPer + EfficientNet-B0/B2 combines FedPer with EfficientNet without the LoRA parameter-efficient fine-tuning technique. LoRA + EfficientNet-B0/B2 performs efficient parameter updates without the FedPer customization layer, focusing on local fine-tuning. The proposed model, FedPer-B0/B2, combines customization (FedPer) with efficient adaptation (LoRA) within the EfficientNet architecture. The EfficientNet-B0 and B2 baselines had notable accuracy (98–99%) but substantial communication costs (20–35 MB). FedPer balances performance and scalability by decreasing the communication costs and preserving near-baseline accuracy. However, LoRA alone (without FedPer) yields 72–75% reduced accuracy despite low communication costs, indicating that LoRA alone is inadequate for robust model generalization. FedPer + LoRA offers an optimal trade-off. The communication costs reduce to 2.53 MB and the accuracy declines to 78.5% for B0. The FedPer + LoRA + EfficientNet-B2 arrangement yielded the best balance, with near-baseline accuracy (98.67%) and a communication cost of 4.12 MB, saving ~ 88% compared to the baseline. The computation efficiency of the proposed model, as depicted in the table, shows that LoRA-based variations without FedPer were computed in 25 min, which seems to be the fastest. FedPer variations without LoRA are the most computationally expensive, taking EfficientNet-B2 75 min. The proposed FedPer + LoRA models show a balance in computation and communication efficiency, with a reduced computation time compared to pure FedPer models.

**Table 11:** Results for the Ablation analysis for the contribution of each component.

Model Variant	Accuracy (%)	F1-Score	AUC	MCC	Kappa	Communication Cost (MB)	Computation Time (min)
EfficientNet-B0	98.1	0.98	0.99	0.98	0.98	20.17	25
FedPer + EfficientNet-B0 (Without LoRA)	98.86	0.99	1	0.98	0.98	15.29	55
LoRA + EfficientNet-B0 (Without FedPer)	72.5	0.68	0.81	0.56	0.55	5.2	25
Proposed FedPer-B0 (FedPer + LoRA + EfficientNet-B0)	78.54	0.76	0.9	0.4	0.41	2.53	45
EfficientNet-B2	99.38	0.99	1	0.99	0.98	34.75	30
FedPer + EfficientNet-B2 (Without LoRA)	99.24	0.99	1	0.99	0.99	29.38	75
LoRA + EfficientNet-B2 (Without FedPer)	74.83	0.7	0.83	0.59	0.58	6.75	25
Proposed FedPer-B2 (FedPer + LoRA + EfficientNet-B2)	98.67	0.99	0.99	0.69	0.7	4.12	50

**Fig. 17:**
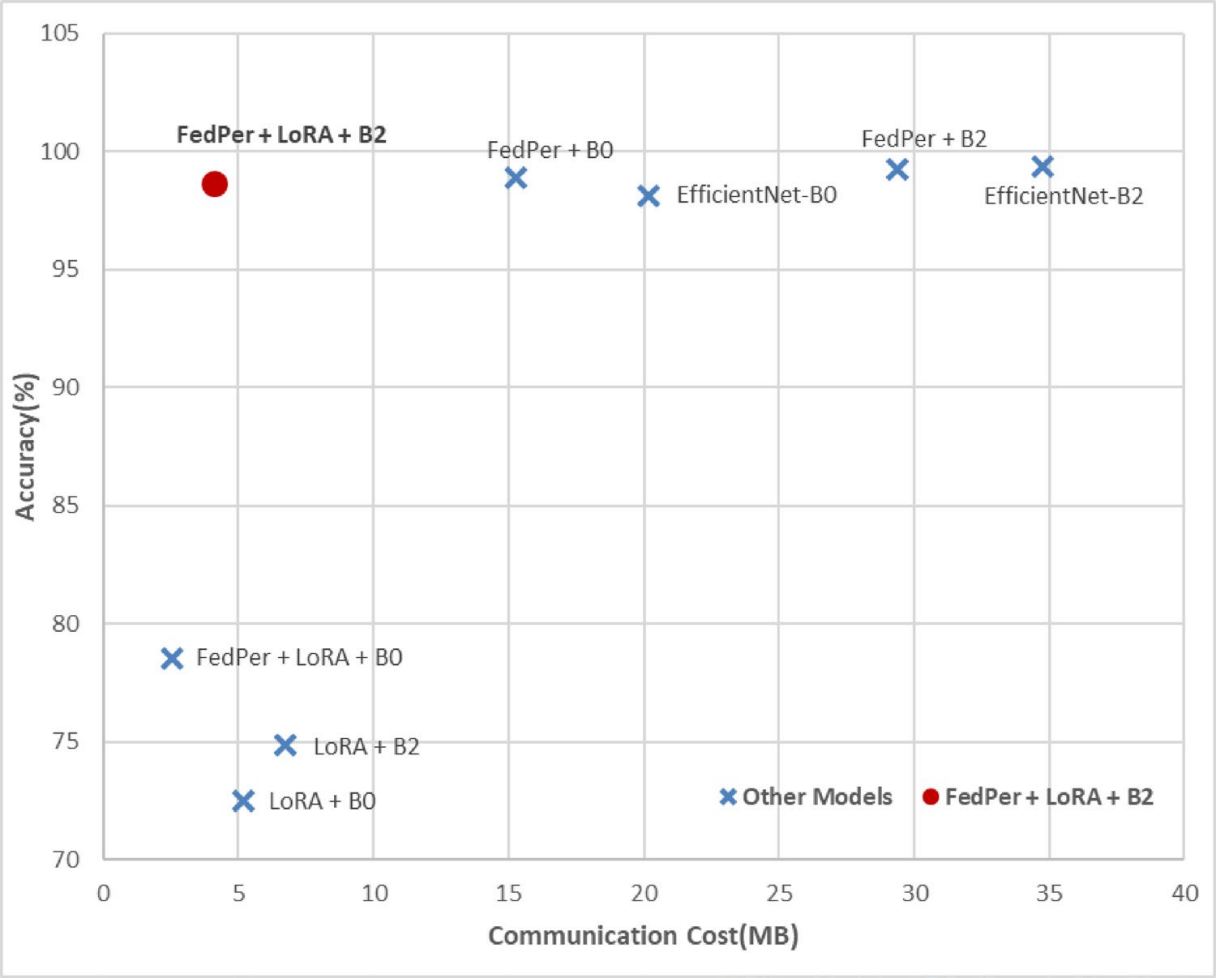
Accuracy Vs Communication Cost Trade-off (Ablation Study).

Figure 17 shows the model variant-specific accuracy-communication cost trade-offs. The ablation analysis shows that the baselines are communication-heavy and unsuited for scaled federated learning, but they perform well. LoRA alone reduces the cost but greatly reduces the accuracy. FedPer and LoRA provided the best balance, particularly with EfficientNet-B2, providing cutting-edge precision with minimal communication overhead. This proves the efficiency and resilience of the proposed method in federated medical imaging.

**Fig. 18:**
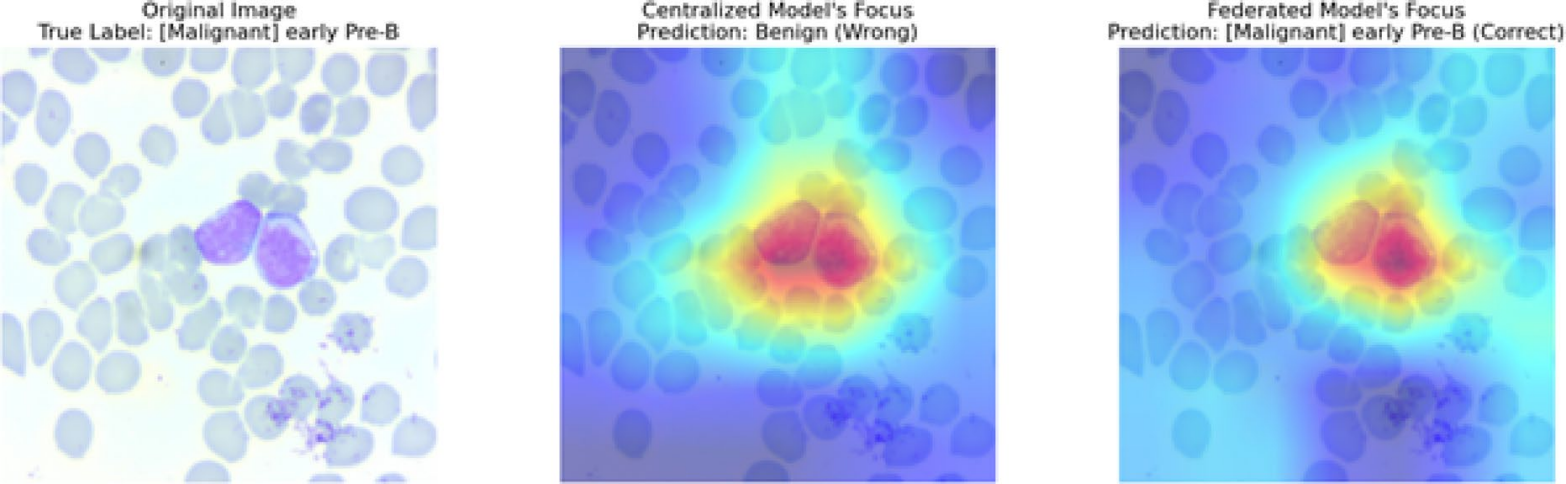
Comparative Explainability using Grad-CAM visualizations (Centralized Vs Federated models).

For this early pre-B ALL case, Grad-CAM visualizations in Fig. 18 showed fundamentally divergent diagnostic techniques between the models. Attention dispersed across non-specific cytoplasmic regions and background artifacts prevented the centralized model from identifying diagnostic nuclear features, such as lymphoblast morphology with a high nuclear-to-cytoplasmic ratio and a dispersed chromatin pattern that pathologists use to identify this ALL subtype. In contrast, FedPer-B2 appropriately focused on the nuclear membranes, chromatin patterns, and small basophilic cytoplasm of malignant lymphoblasts. Owing to its focus on WHO-defined diagnostic criteria (2016 categorization), federated training may improve hematopathological feature learning across institutions by decreasing site-specific staining artifact biases that may impact centralized models. This instance shows how the data variety of federated learning may improve rare leukemia subtype morphological feature selection, where centralized models overfit to institutional trends.

**Fig. 19:**
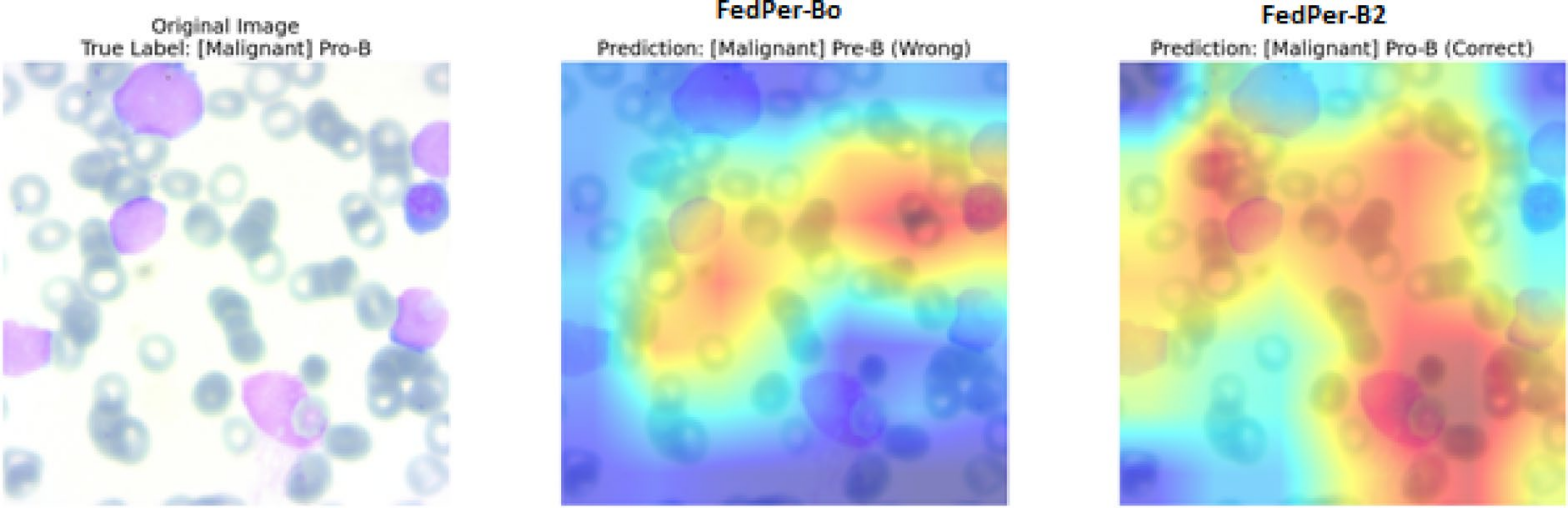
Comparative Explainability using Grad-CAM visualizations (FedPer-B0 Vs FedPer-B2).

The attention maps show that FedPer-B0 and FedPer-B2 have significantly different diagnoses for this Pro-B ALL instance as shown in Fig. 19. Both models accurately diagnosed malignancy although FedPer-B0 misclassified the subtype as Pre-B ALL because of its focus on cytoplasmic traits rather than nuclear features of Pro-B ALL (such as open chromatin and prominent nucleoli). FedPer-B2’s proper classification was connected with careful attention to diagnostic nuclear characteristics, specifically nuclear membrane abnormalities and chromatin distribution patterns pathognomonic for Pro-B ALL according to the WHO 2022 criteria. In federated settings, the B2 architecture’s increased representational capacity allows more precise subtyping by focusing on diagnostically critical cellular features, while the B0 model’s limited capacity over-reliance on less specific morphological characteristics.

**Fig. 20:**
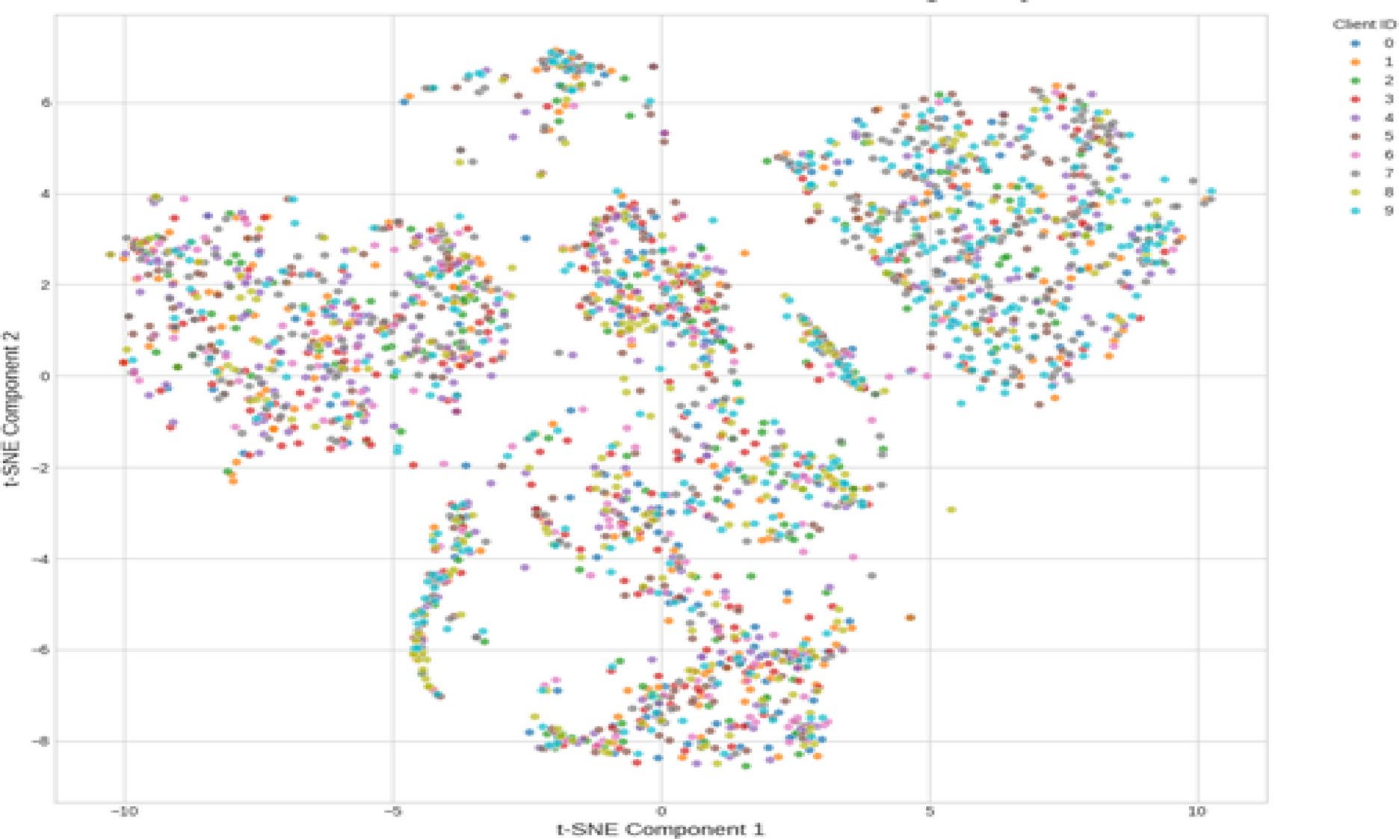
t-SNE Client Data (Non-IID data) Heterogeneity.

The t-SNE projection in Fig. 20 illustrates clinically relevant non-IID heterogeneity in the proposed federated learning configuration employing Dirichlet partitioning (α = 0.7). Client 2 had a substantial Pre-B skew (47.4%), and Client 9 was dominated by Pro-B cases (37.1%), resembling paediatric or adult leukemia facilities. Clients 4, 5, and 7 formed center clusters with balanced subtype distributions, indicating general-purpose hospitals, whereas Clients 0, 3, and 6 had higher Early Pre-B prevalence, indicating comparable patient demographics. These patterns support the biologically realistic institution-level feature coherence. Large inter-cluster distances underscore the federated problem of learning during distributional shifts, as indicated by performance disparities (e.g., Client 5 AP = 0.616 vs. Client 6 AP = 0.937) when utilizing lightweight models such as FedPer-B0. FedPer-B2’s increased alignment and robustness across dispersed clusters demonstrate its architectural benefits in addressing multi-institutional variance. The t-SNE graphic confirmed the clinical realism and value of the setup as a rigorous tailored federated learning testbed.

## Conclusion

FedPerLoRA-Health, a unique and communication-efficient federated learning architecture for customized leukemia diagnosis, was presented in this paper. The suggested method uses LoRA modules in the EfficientNet-B0 and B2 backbones to fine-tune tailored local models without compromising client data privacy. FedPerLoRA-Health minimizes the communication cost per round in real-world healthcare settings with bandwidth limits by freezing most model parameters and training only lightweight LoRA adapters and private classification heads. FedPerLoRA-Health outperformed the baseline centralized and federated models in terms of convergence speed and communication efficiency on the Blood Cell Cancer (ALL) dataset. Private heads and LoRA adapters offer tailored training that captures client-specific data distributions, addressing non-IID data problems in medical institutions. Even though the proposed model addresses the mentioned research gaps, it has few limitations such as real-world deployment feasibility and multi-institutional trials. While FedPerLoRA-Health has shown promising results in communication efficiency and tailored leukemia diagnosis, various practical extensions can improve its application and robustness. The ~ 88% reduction in communication overhead implies a proportionate reduction in energy usage. This research sets a foundation for communication efficiency, however, future work will quantify energy savings and hardware-level optimizations for clinical edge devices provided by parameter reduction. Personalization can be increased by tailoring LoRA module complexity to client-specific hardware capabilities for effective deployment over a heterogeneous network of devices.

## Data Availability

The data that support the findings of this study are available from the corresponding author upon reasonable request.
